# Identification of divergent *Toxoplasma* Nuclear Pore Complex components highlights speciation of mRNA export machinery

**DOI:** 10.1101/2025.08.27.672535

**Published:** 2025-08-27

**Authors:** Pravin S. Dewangan, Shannon R. Dohr, Jackson Trotter, Michael L. Reese

**Affiliations:** 1 Department of Pharmacology, University of Texas, Southwestern Medical Center, Dallas, TX USA; 2 Department of Biochemistry, University of Texas, Southwestern Medical Center, Dallas, TX USA

**Keywords:** Evolutionary cell biology, Nuclear pore complex, Apicomplexa, Parasitology, BIOLOGICAL SCIENCES: Microbiology

## Abstract

**Background:**

A hallmark of the eukaryotic cell is the regulated transport between the nucleus and cytoplasm, which is mediated by a multi-subunit protein assembly called the nuclear pore complex (NPC). While its overall architecture has been preserved across eukaryotes, variations in NPC structure appear to have tuned its function in different organisms. Outside of a handful of model systems, the NPC has not been comprehensively studied. This is particularly true of species that are not closely related to well-studied models, such as apicomplexan parasites. Indeed, the evolutionary divergence of Apicomplexa has complicated facile prediction of these proteins in these organisms. Because of this, the NPC components remain largely unidentified, and therefore NPC cellular function in Apicomplexa is poorly understood.

**Principal Findings:**

Here we identified, experimentally validated, and functionally characterized protein components of the NPC in the apicomplexan parasite *Toxoplasma gondii*. By combining proximity biotinylation with careful bioinformatic analysis we identified 15 previously uncharacterized proteins that localize to the *Toxoplasma* NPC. We demonstrated 7 of these proteins are essential to parasite replication. Importantly, we defined components of the mRNA export machinery, as well as subunits required for the stability and/or assembly of specific NPC subcomplexes. Consistent with the evolutionary distance between *Toxoplasma* and well-studied models, the majority of our newly validated NPC components show no clear homology to NPC proteins in yeast, animals, or plants. Moreover, we demonstrated that the *Toxoplasma* mRNA export machinery has a distinct composition from other well-established systems. Intriguingly, several well-defined domains the TREX-2 complex that are essential in other systems are missing from the *Toxoplasma* genome. In contrast, others, such as Centrin-3, have been conserved in *Toxoplasma*, but are not required for mRNA export in the parasite.

**Conclusion:**

Our work highlights the distinct composition of multiple subcomplexes of the *Toxoplasma* NPC and paves the way for future studies to provide high-resolution structural information on the parasite’s unusual NPC architecture.

## Introduction

**Figure 1:**
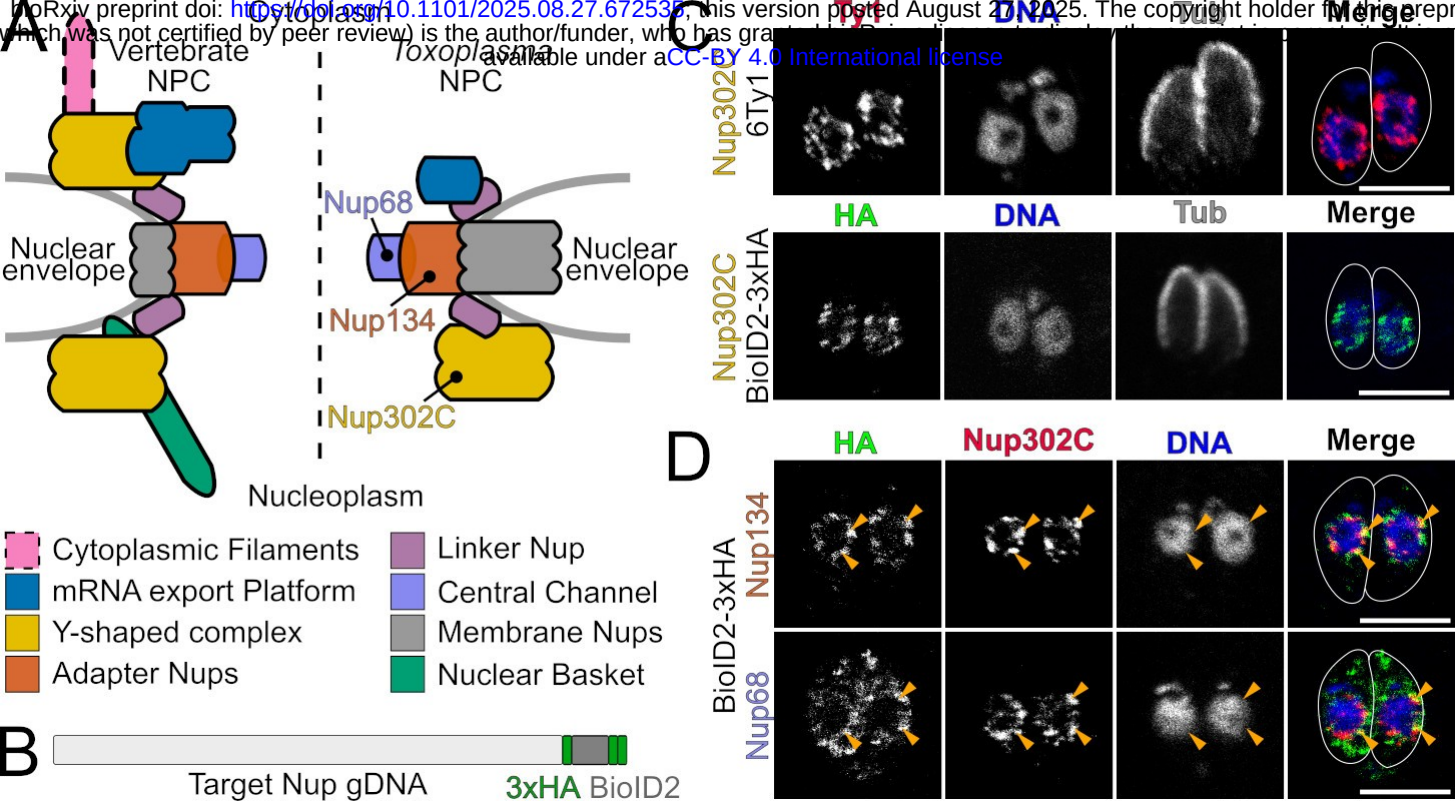
*Toxoplasma* Nup BioID strains localize to NPC. (A) Cartoon overview highlighting differences in the well-studied vertebrate (left) and *Toxoplasma* (right) NPC architectures. The three proteins we use as BioID baits and their respective subcomplexes are indicated by color. (B) Each Nup was endogenously tagged to express an in-frame C-terminal 3xHA-BioID2. (C) In all candidate strains, we use Nup302C^6xTy1^ as a marker for the NPC. (D) Validation of Nup68 and Nup134 BioID. Note that the yeast and human orthologs of Nup68 also show dual NPC and cytoplasmic localization [12,43].

The nuclear pore complex (NPC) is one of the largest macromolecular assemblies in a eukaryotic cell, and serves as the gateway through which RNA and proteins are trafficked between the nucleus and cytoplasm [1]. In all organisms, the NPC is composed of a series of concentric rings with 8-fold symmetry [2–4]. The NPC is well-conserved across eukaryotic phyla, and structural studies have revealed a common core architecture in plant, fungal, and animal species [5–10]. The NPC rings are composed of multiple biochemically-defined subcomplexes, comprised of proteins called nucleoporins (Nups) [11–14] (Figure 1A), and it is these subcomplexes that are most highly conserved. Indeed, the major components of the NPC have been suggested to have been conserved in the last common eukaryotic ancestor [15]. Nevertheless, while its core structure is preserved, the NPC varies across different lineages [16,17]. Nowhere is this more evident than in apicomplexan parasites like *Toxoplasma gondii*. A recent cryo-electron tomography study compared the in-cell architectures of the NPC from yeast, human, and *Toxoplasma* cells and found that the parasite’s NPC is unusually asymmetrical, having lost the Y-shaped complex from its cytoplasmic face [7]. In addition, while the *Toxoplasma* nuclear basket could not be resolved, there appeared to be a much larger membrane-anchored core in the parasite NPC [7] (Figure 1A).

In addition to *Toxoplasma*, the phylum Apicomplexa includes the causative agents of a number of the world’s most devastating diseases, such as the *Plasmodium* species that cause malaria. *Toxoplasma* and other apicomplexan parasites are members of the Alveolate superphylum and are highly divergent from well-studied animal, fungal, and plant models. Because of this, many proteins that are well-conserved in other organisms are difficult to identify by standard bioinformatic methods. This has stymied research into many basic cell biological processes, including the components of the apicomplexan NPC. To date, a handful of studies have validated 11 proteins as NPC components in *Plasmodium* [18,19] and 8 in *Toxoplasma* [20,21]. In yeast and humans, the NPC is composed of multiple copies of ~30 proteins [22,23], suggesting that many NPC components in Apicomplexa have yet to be identified and experimentally validated. Components of the NPC are called nucleoporins or Nups and have historically been numerically named based on their apparent molecular weights on an SDS-PAGE (*e.g.* “Nup57”). This means that orthologous proteins rarely share a numerical name and non-orthologous proteins in different organisms can have the same number. To avoid confusion, we will use a numerical Nup nomenclature only when referring to Nups from model organisms such as yeast and those few parasite proteins that have been previously named.

Here we integrated proximity biotinylation with carefully curated bioinformatic analysis to identify and validate 15 additional *Toxoplasma* Nups. We went on to demonstrate that, as in other organisms, the *Toxoplasma* NPC is robust to deletion of individual Nups, in that only a handful of the proteins we validated as Nups are essential. We also identified components of two NPC-associated subcomplexes that are required for the export of mRNA. Finally, using markers for two distinct NPC subcomplexes that localize to opposing faces of the nuclear envelope, we identified Nups required for the assembly of NPCs in *Toxoplasma.* Together these data provide the most comprehensive functional characterization of the components of the NPC in an apicomplexan organism and will enable future indepth cellular and biophysical studies.

## Results

### Coupled bioinformatic analysis and proximity biotinylation identifies components of the Toxoplasma nuclear pore complex

In diverse organisms where the NPC has been most thoroughly investigated, there are approximately 30 Nups that comprise the NPC [22,23]. Previous efforts have predicted components of the *Toxoplasma* NPC by bioinformatic analysis [24] and immunoprecipitation/mass spectrometry [20,21]. To date, 8 of these proteins have been validated as localizing to the NPC [20,21]. Our first goal was to expand this number. Because the NPC is highly stable, we reasoned that proximity biotinylation [25,26] would be an excellent method to identify new nucleporins in *Toxoplasma*. Because we saw toxicity when fusing some proteins to TurboID [27], we decided to use the smaller BioID2 [28] protein. As probes, we chose 2 validated *Toxoplasma* Nups and 1 additional Nup after validating it’s localization (Figure 1). These proteins are predicted by their homology to be associated with three different NPC subcomplexes (Figure 1; TgNup68 – Central channel; TgNup134 – Adapter; TgNup302C – Y-shaped complex). Each of these proteins faithfully localized to the *Toxoplasma* NPC when endogenously tagged at their C-termini with a BioID2–3xHA fusion. Note that while Nup134 and TgNup302 had been previously validated, TgNup68 had not been previously localized to the NPC. We found that TgNup68 showed a dual NPC and cytoplasmic localization, similar to its yeast ortholog, ScNsp1 [12,29]. For proximity biotinylation experiments, parasites for each BioID strain and the untagged parental strain were grown in 150 μM biotin for 40 h before lysis and purification on streptavidin resin. Proteins were identified by mass spectrometry and each Nup-BioID sample was compared to the untagged parental strain negative control (Supplemental Data S1).

We first ranked candidates by the number of datasets with a minimum 2-fold change over the parental control. We further filtered these candidates based on predicted localization to other organelles based on potential targeting signals [30], domains functioning distinct from the NPC (*e.g.* transcription factors), and published subcellular fractionation data [31]. Finally, we closely examined predicted secondary structure and domains to identify patterns and sequence motifs that are often found in Nups (Supplemental Figures S2-S3). Encouragingly, this list included 13 of the previously predicted Nups, 5 of which had been previously validated. We also added TG*_310610 to this list, as it had been previously predicted as a Nup, but had been left unvalidated. This left us with 25 candidates to interrogate as potential Nups.

**Figure 2:**
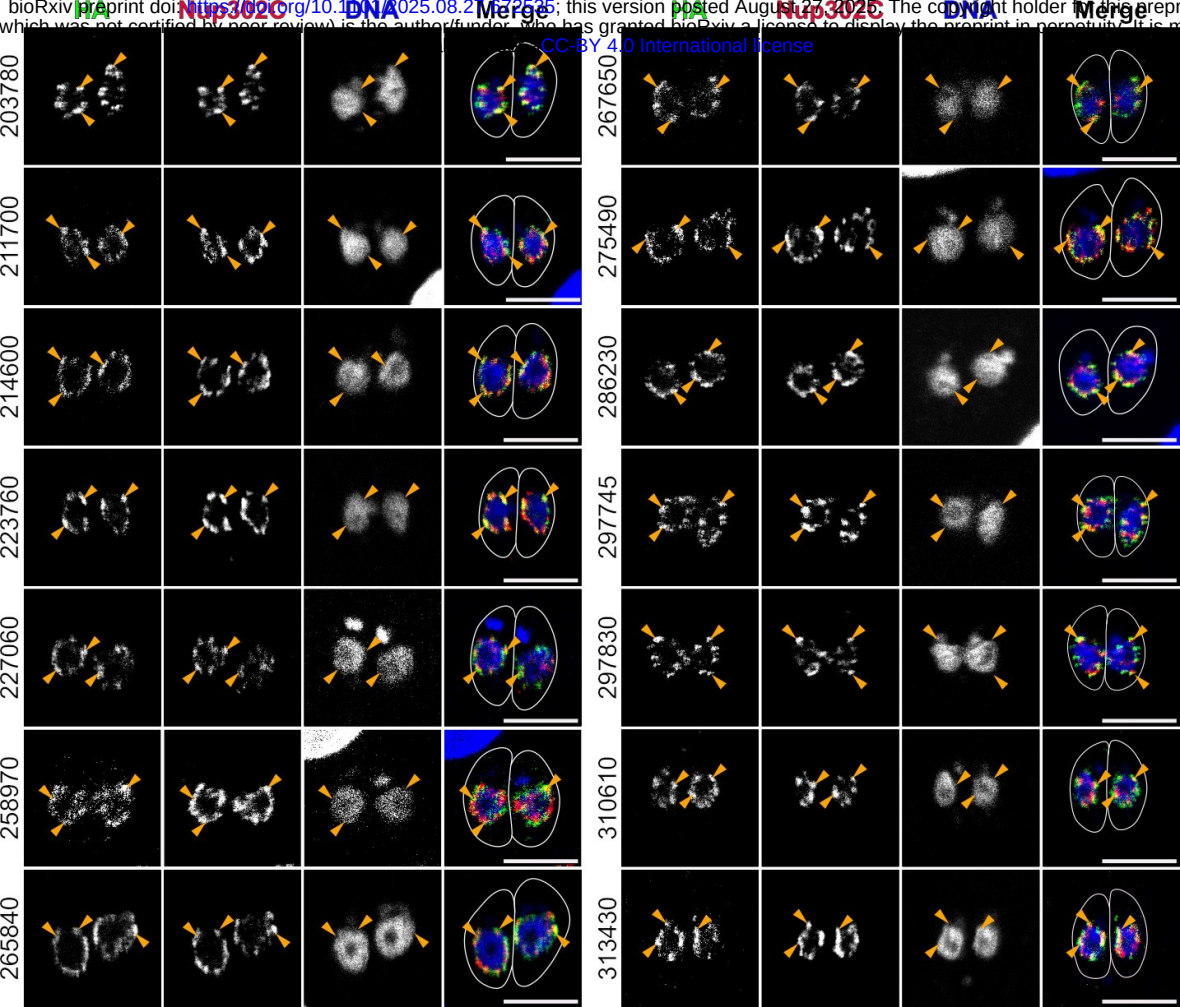
Validation of NPC localization for candidate Nups. Intracellular parasites in which the indicated candidates (for clarity, only the numerical identifiers are indicated, *i.e.*, TG*_######) had been tagged with AID-3xHA were stained with antibodies recognizing HA (green), Ty1 (red; Nup302C), and *Toxoplasma* β-tubulin (not shown; used to create outline). DNA was stained with Hoechst (blue). Images are 1 airy-unit confocal slices (~0.75 μm). Clear NPC-associated staining is noted with orange arrowheads. All scale bars are 5 μm.

We next sought to validate the localization of these proteins to the NPC. We successfully generated *Toxoplasma* strains in which 18 of 25 of the candidates expressed a C-terminal tag comprising an AID degron [32] and 3xHA epitope tag in the background of a strain in which the TgNup302C protein was fused to a C-terminal 6xTy1 epitope. These strains allowed us to demonstrate that 14 of our 18 candidates localized to the NPC (Figure 2 & Supplemental Figure S4), and were therefore bona fide Nups. Notably, we observed no convincing NPC localization for 1 of the previously predicted Nups (TG*_248500/“TgNup37” [20]; Supplemental Table S5), in spite of its strong homology to yeast Nup57.

### Most Toxoplasma Nups have no clear orthologs in model organisms

The individual protein components of the NPC appear to be remarkably widely conserved among eukaryotes, as clear orthologues to yeast proteins have been found in protists such as trypanosomes [15,33,34]. We attempted to similarly predict orthologs of each of the validated *Toxoplasma* Nups using a combination of sequence (BLAST [35]; the PANTHER domain database [36]) and structure-based (Foldseek [37]) methods (Supplemental Figures S2-S3, Table S5). We also included in our analysis Nups that remain unvalidated due to experimental difficulty, but that nevertheless have strongly predicted orthologs. The strongest support by all methods are for proteins with well-folded domains, including the previously predicted orthologs of Sec13 and Rae1 [20,24]. We also identified TG*_211700 as a likely ortholog of yeast Sac3, the core scaffold of the TREX-2 RNA export machinery [38,39]. Two major motifs found in many Nups are β-propellar WD40 domains and the so-called FG repeats found in many of the proteins that line the pore of the NPC [40,41]. While many of the *Toxoplasma* Nups contain these motifs (Supplemental Figures S2-S3), their existence alone is not sufficient to predict orthologs. For instance, the only significant region of sequence similarity found by BLAST in most *Toxoplasma* FG repeat proteins are the FG repeats themselves, which are by their nature low complexity. Similarly, the predicted WD40 domains of many of the *Toxoplasma* Nup match to non-Nup proteins in other organisms when searched (Supplemental Table S5). Foldseek searches are limited by the depth of sequence available to identify evolutionary relationships. We lack a robust set of sequences for organisms that link Apicomplexa to the last-eukaryotic ancestor, so AlphaFold models are often of much lower confidence than for other organisms. All together, we confidently identified orthologs to only 7 *Toxoplasma* Nup proteins (Reciprocal BLAST/Foldseek with e-value <10^3^) Therefore, current tools for bioinformatic analyses appear ill-suited to inform on the potential functions of *Toxoplasma* Nups.

### The Toxoplasma nuclear pore complex is robust to nucleoporin loss

**Figure 3:**
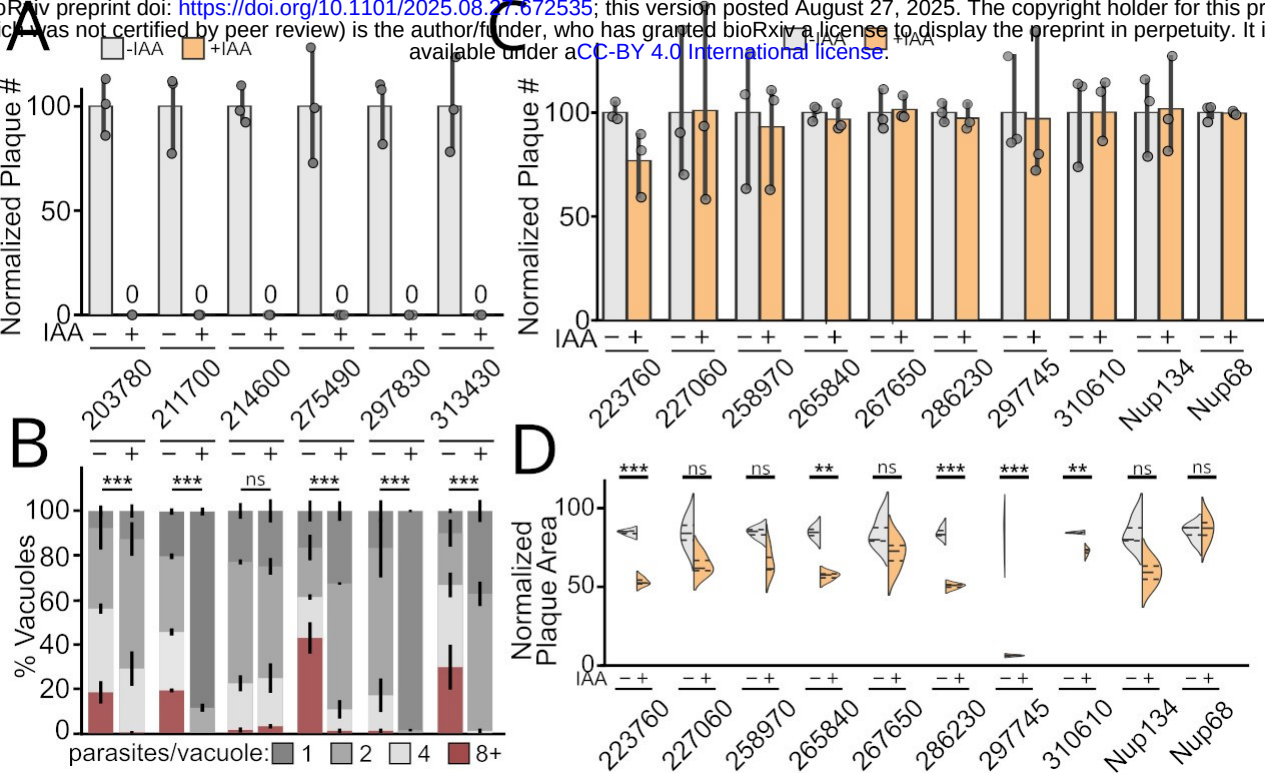
The *Toxoplasma* NPC is robust to loss of individual proteins. (A) Growth in +IAA media completely blocked the ability of 6 Nups to form plaques on a fibroblast monolayer. (B) The same strains were grown for 18 h post-infection ±IAA and showed varying levels of block in replication (see also Supplemental Figure S6). ***, p<0.001 by Kolmogorov-Smirnof test. Quantification of 100 vacuoles from per n=3 biological replicate. (C) Other Nups showed no significant loss in plaque efficiency when grown in +IAA media. (D) Quantification of plaque area indicates a fitness cost upon depletion of a subset of these non-essential Nups. n=3 biological replicates. **, p<0.01; *** p<0.001 by unpaired two-tailed Student’s t-test.

The NPC is an enormous protein complex that carries out an essential cellular function. Nevertheless, the NPC appears remarkably robust to loss of individual components. For instance, in yeast, only 12 of 33 Nups are essential [42]. To test the fitness costs associated with loss of the Nups we identified above, we used the auxin-inducible degron system. Parasites were plated on fibroblast monolayers in media with or without the auxin indole-3-acetic acid (IAA), and allowed to grow for 7–10 days. This allows for multiple rounds of invasion, growth, and lysis to occur, producing plaques where the monolayer has been disrupted. We tested the fitness cost of the 14 Nups we had validated, as well as TgNup68 and TgNup134, which have not been previously phenotypically characterized. We found that 6 of the Nups were essential (TG*_203780, TG*_211700, TG*_214600, TG*_275490, TG*_297830, TG*_313430); induced degradation of these proteins resulted in a complete loss of plaque formation (Figure 3A). We also found that degradation of an additional 4 Nups (TG*_223760, TG*_265840, TG*_297745, TG*_310610) resulted in significant fitness cost, though the proteins were not essential (Figure 3B).

A reduction in plaque number can be due to a block in replication or in motility and invasion. Some Nups, such as human Nup62 (TgNup68), have additional localization associated with functions independent of their roles in nucleocytoplasmic transport [43,44]. In addition, some FG repeat Nups have recently been implicated as moonlighting as barrier-forming proteins at the base of the cilium in animals [45–47]. The *Toxoplasma* invasion machinery is thought to have evolved from the eukaryotic cilium [48]. We therefore sought to determine whether the essentiality of the above *Toxoplasma* Nups was due to a block in replication or at another step in the lytic cycle. We grew each of the 6 essential Nup-AID strains in ±IAA media for 16 hours and quantified the number of parasites per vacuole (Figure 3C). We found that loss of 2 of the Nups (TG*_211700 and TG*_297830) caused a severe block in replication, in which the majority of parasites failed to divide even once (Figure 3C). 3 of the validated Nups (TG*_203780, TG*_275490, TG*_313430) showed significant defects in replication at 16 h (Figure 3C), and by 24 h growth in IAA exhibited aberrant morphologies, demonstrating that they were nonviable (Supplemental Figure S6A,B). Induced degradation of the last Nup, TG*_214600, showed no significant effect on replication after 16 h growth in +IAA. However, these parasites also failed to replicate past 24 h in +IAA medium, again suggesting nonviability (Supplemental Figure S6). Taken together, these data are consistent with essential roles in NPC function rather than any specialized moonlighting function in invasion or motility.

### The Toxoplasma Nups TG*_211700 and TG*_297830 are required for mRNA export from the nucleus

**Figure 4:**
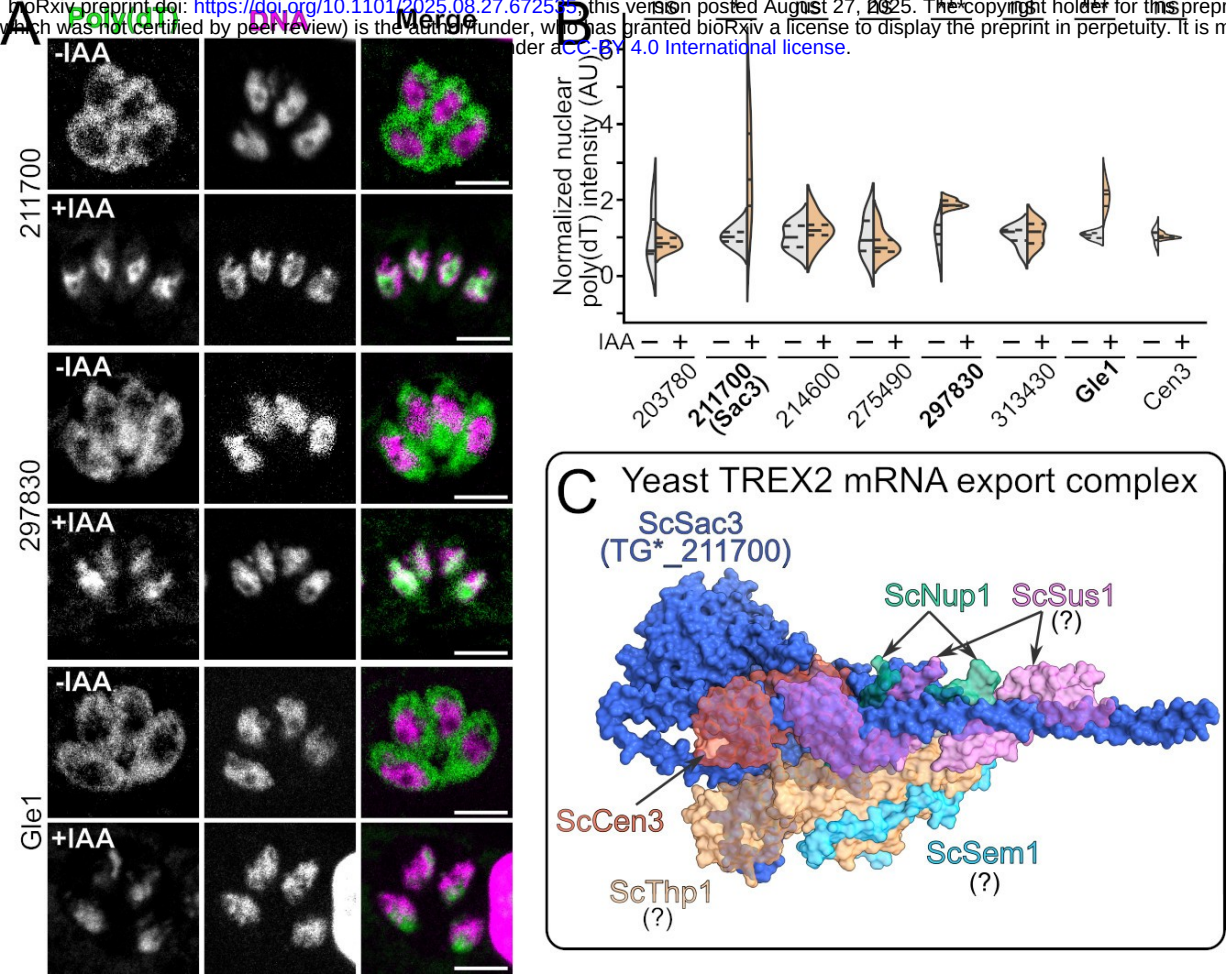
Identification of *Toxoplasma* Nups required for mRNA export. (A) Confocal slices of parasites that were allowed to infect overnight and then grown in +IAA media for 2 h before fixation. mRNA was stained by FISH with poly(dT) oligo (green) and parasites were counterstained with Hoechst (magenta). (B) Quantification of images from (A). A minimum n=20 vacuoles were quantified per n=6 biological replicates. ns, not significant; ***, p<0.001; two-tailed unpaired Student’s t-test. (C) Model of the yeast mRNA export complex demonstrates well-folded domains for each of the components. Orthologs of ScNup1 and ScCen3 appear present in *Toxoplasma* but appear uninvolved in mRNA export. Sac3 (blue) is the only other component we have been able to identify *Toxoplasma* (missing components are indicated with a question mark).

One of our candidates had a predicted role in mRNA export. TG*_211700 has significant homology to SAC3, which forms the central scaffold of the TREX-2 nucleoplasmic mRNA export machinery (PDB: 4MBE and 8U8D; PTHR12436 – e-value: 4×10^−37^; Supplemental Table S5) [49,50]. As mRNA export is an essential function of the NPC which would be expected to cause an immediate block in replication if disrupted, we tested whether induced degradation of TG*_211700 and the other essential nucleoporins affected nuclear mRNA export in *Toxoplasma*. We expected mRNA export to be disrupted immediately upon loss of the proteins involved, so tested a brief 2 h incubation with IAA. Parasites were allowed to infect host cells in normal media for 6 h, and then changed to ±IAA media for another 2 h before fixation. Mature mRNA was stained by fluorescent *in situ* hybridization (FISH) using a fluorescein poly(dT) oligonucleotide. Parasites were counterstained with Hoecsht, and the nuclear poly(dT) signal for each parasite was quantified. Consistent with a predicted role in mRNA export TG*_211700 showed a significant increase in mRNA build up in the nucleus upon growth in +IAA (Figure 4A,B). Only one other candidate, TG*_297830, also showed a clear block in mRNA export upon growth in +IAA. This protein, like TG*_211700, also showed the fastest-onset replication defect we observed (Figure 3B). TG*_297830 does not have a predicted nuclear localization signal, so is likely present on the cytoplasmic side of the NPC. The secondary structure profile of TG*_297830 is similar to that of yeast ScNup82 (Supplemental Figure S2). ScNup82 forms the core of the mRNA export complex on the cytoplasmic face of the yeast NPC and, like TG*_297830, is essential for mRNA export [51]. These observations strongly argue that TG*_297830 is the *Toxoplasma* ortholog of ScNup82.

Our data also suggest that TG*_211700 is, indeed, a functional ortholog of SAC3. Unlike many proteins associated with the NPC, the TREX-2 complex is composed of proteins that have well-defined globular folds (Figure 4C). We reasoned this should facilitate prediction of other members of the TREX-2 complex in *Toxoplasma*. However, searching using both sequence-based (BLAST, Hmmer) and structural (Foldseek) methods, we were unable to find significant matches (evalue <10^−3^) to the TREX-2 components Thp1, Sus1, or Sem1 (Supplemental Table S5). However, other proteins can play a role in supporting TREX-2 function. Centrin-3, also called Cdc31 in yeast, helps anchor the TREX-2 complex in other organisms [52,53]. Centrin-3 has a previously described ortholog in *Toxoplasma* [54]. We therefore created a strain in which TgCentrin-3 (Cen3) has been tagged with AID-3xHA, as with our other candidates (Supplemental Figure S7). We tested this strain for a defect in mRNA export, but saw no significant difference between ±IAA conditions (Figure 4), suggesting that TgCen3 is not a central player in this process. We also searched for other members of the cytoplasmic mRNA export platform in the *Toxoplasma* genome. We identified TG*_259172 as sharing significant homology with yeast Gle1 (PTHR12960 – evalue: 3×10^−37^; Supplemental Table S5), a protein essential for both mRNA export and translation termination in other organisms [55,56]. Consistent with this observation, when we created a TgGle1^AID−3xHA^ strain, we found that it localized to the NPC (Supplemental Figure S7) and induced degradation of the protein caused a striking buildup of mRNA in the nucleus (Figure 4). These data indicate that TgGle1 is indeed an ortholog of the yeast protein and has critical role in mRNA export in *Toxoplasma*. Together our data highlight that, like the NPC itself, the mRNA export machinery in *Toxoplasma* has conserved central components, but has diverged in its specific architecture.

### Identification of Nups required for NPC stability or assembly

**Figure 5:**
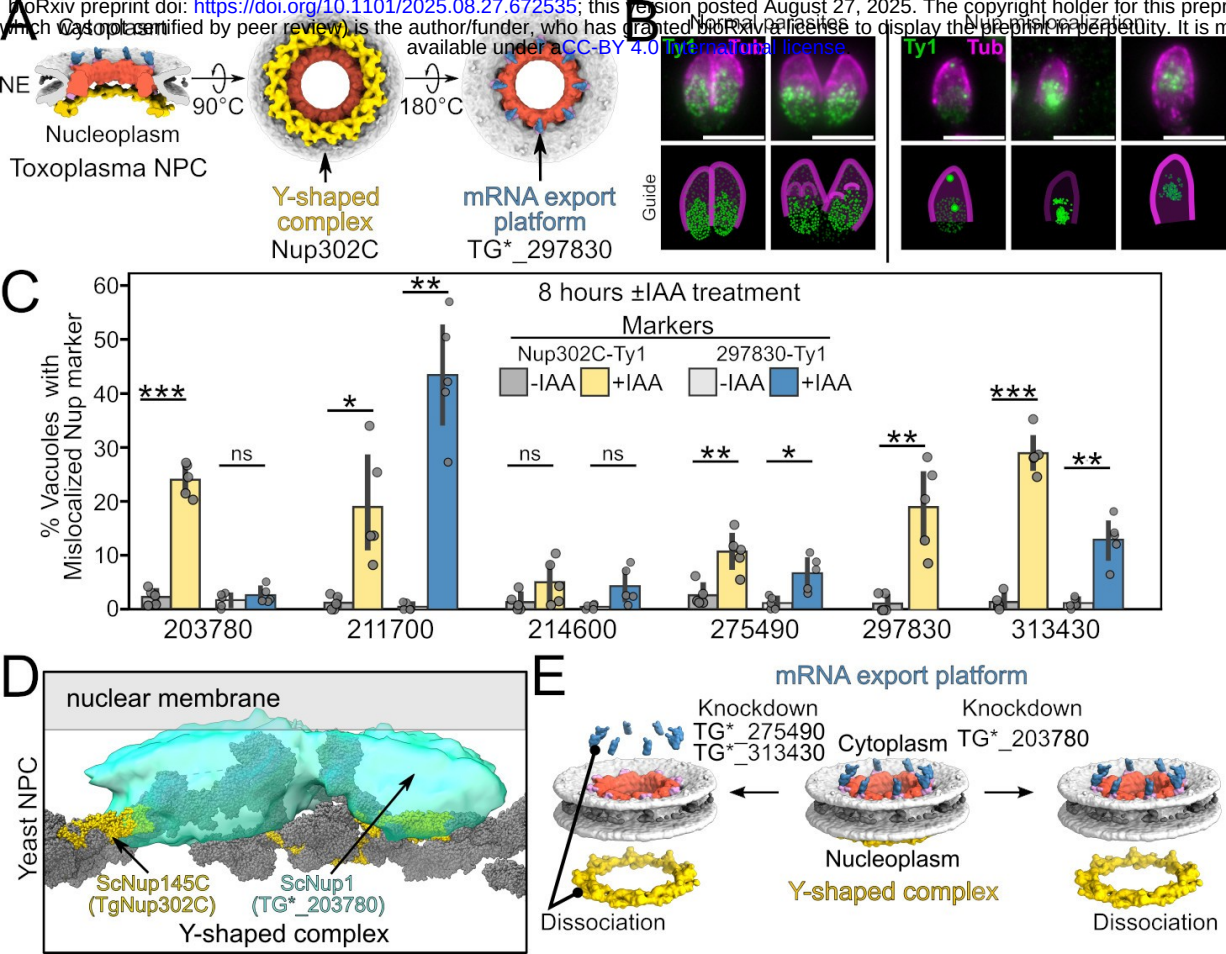
Mislocalization of markers of NPC subcomplexes identifies Nups likely involved in assembly of NPC. (A) Model of the *Toxoplasma* NPC from [7] indicates likely localization of Nup302C in the nucleoplasmic Y-shaped complex (yellow) and TG*_297830 in the cytoplasmic mRNA export platform (blue). (B) Guide for phenotyping using wide-field micrographs. Note nuclear punctate staining of marker Nups (green) centered in tubulin outline (magenta). (C) Quantification of mislocalization of the indicated marker in parasites that had been grown for 8 h ±IAA post-infection. Note that because TG*_297830 is used as a marker, we quantified only mislocalization of Nup302C. (D) CryoEM model of the yeast Y-shaped complex shows likely localization of TgNup302C (yellow) and ScNup1 (cyan volume), which helps anchor the Y-shaped complex. (E) Cartoon summary of data indicating knockdown of certain Nups affects dissociation of specific subcomplexes from the *Toxoplasma* NPC.

Another way in which a nucleoporin might be essential is by stabilizing existing NPCs or by facilitating the biogenesis of new complexes. We therefore tested whether inducing degradation of any of the 6 essential Nups caused mislocalization of our established NPC marker, Nup302C^6xTy1^, which is part of the Y-shaped complex on the nucleoplasmic face of the NPC (Figure 5A). We also tested for mislocalization of TG*_297830, as our data suggest it is part of the cytoplasmic mRNA export platform (Figures 4, 5A). To this end, we created parasites in which the endogenous TG*_297830 protein was expressed as a C-terminal fusion to 6xTy1 and tagged the other 5 essential Nups with AID-3xHA in this background. Parasites were allowed to infect host cells for 2 h in normal media and then switched to ±IAA media for another 8 h before fixation. In all cells grown in -IAA, both marker Nups showed characteristic punctate staining on the nuclear envelope (Figure 5B). Loss of 5 Nups, however, caused mislocalization of TgNup302C (Figure 5C). Similarly, loss of 3 Nups caused aggregation and mislocalization of TG*_297830 (Figure 5C).

Consistent with the rapid block of replication upon their loss, TG*_297830 and TG*_211700 (TgSac3) showed the strongest penetrance of the mislocalization phenotypes (Figure 5C). Given the substantial effects of their loss on Nup localization at 8 h (Figure 5C), we sought to ascertain which phenotype associated with these proteins was primary (mRNA export or NPC stability). To this end, we tested whether loss of either protein caused a significant defect in Nup marker localization over the same short 2h time period we used for the mRNA export assay. We also tested whether loss of TG*_297830 affected TG*_211700 localization to the NPC at this time point. For all markers tested, loss of either protein caused no significant disruption to the NPC architecture in this timeframe (Supplemental Figure S7C). Our data therefore suggest that both TG*_211700 and TG*_297830 directly function in the export of mRNA in *Toxoplasma* and the severe mislocalization of markers at later time points is a secondary effect of this function.

TG*_275490 and TG*_313430 also showed a modest penetrance, in which 5–20% of vacuoles showed mislocalization of both markers. Intriguingly, degradation of TG*_203780 resulted in the specific mislocalization of nucleoplasmic-facing Nup302C, but not cytoplasmic-facing TG*_297830 (Figure 5C). Notably, ScNup1 is an FG repeat protein that is part of the nuclear basket and anchors the Y-shaped complex to the nuclear membrane and the rest of the NPC in other organisms (Figure 5D). TG*_203780 is also an FG repeat protein, and therefore may be functioning equivalently to ScNup1 at this site. Notably, the *Toxoplasma* Y-shaped complex is found only on the nucleoplasmic face of the NPC [7] and degradation of TG*_203780 resulted in the specific mislocalization of Nup302C. Taken together, these data strongly suggest TG*_203780 is a functional ortholog of ScNup1 and a potential component of the *Toxoplasma* nuclear basket. Because degradation of TG*_275490 and TG*_313430 caused mislocalization of markers found on opposing faces of the NPC (Figure 5C,E), we reason that these proteins are part of the central adapter complex, which is consistent our predictions based on secondary structure (Supplemental Figure S2–3, Table S5).

## Discussion

**Figure 6:**
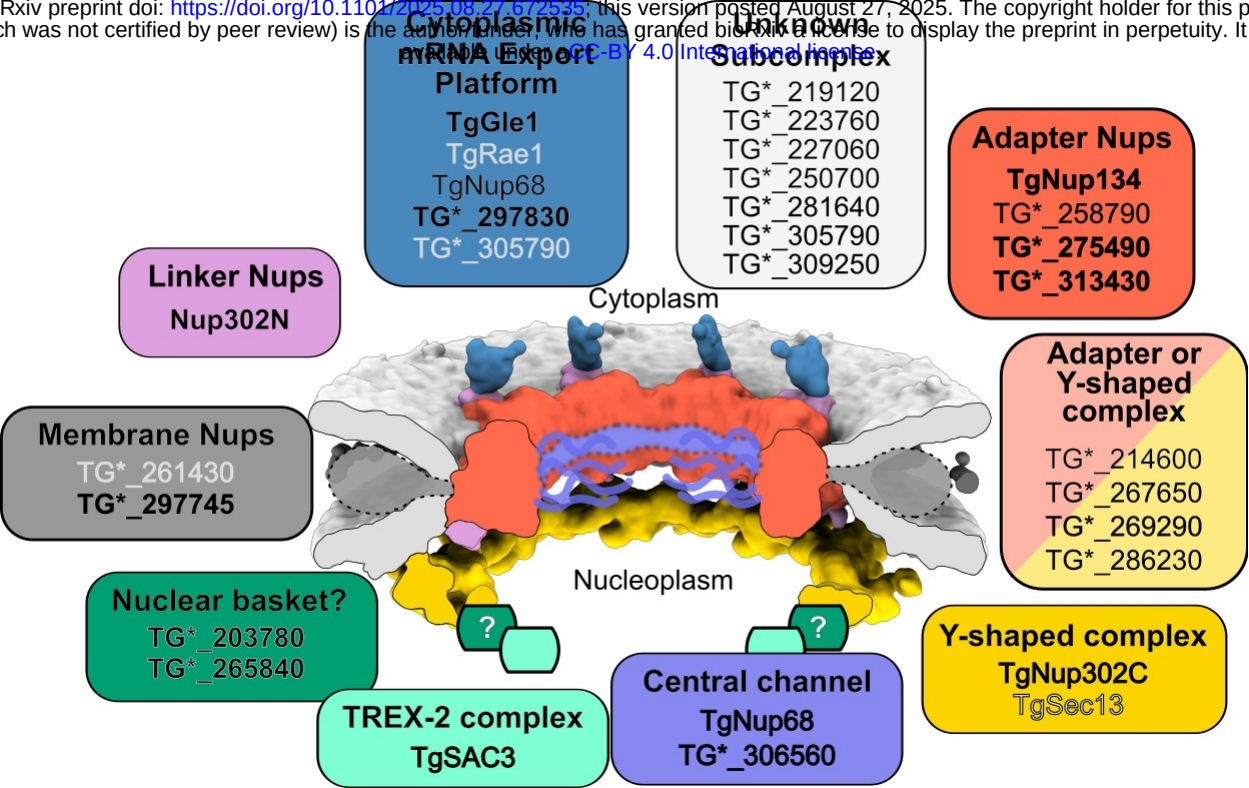
Model *Toxoplasma* NPC subcomplexes – Model of the *Toxoplasma* NPC from cryoET maps [7] colored by subcomplex. Note that the densities for membrane Nups and central channel are poorly resolved, and have been replaced with cartoon representations. Nups have been grouped according to predicted subcomplex membership. Validated Nups in black text and bolded when we have high confidence in their membership in a given subcomplex, while predicted Nups that remain unvalidated are in gray text.

The *Toxoplasma* NPC has a distinct architecture from other model organisms, and nucleoporins that comprise this structure have been difficult to predict. Using a combination of proximity biotinylation and bioinformatic analysis, we have greatly expanded the functional catalog of *Toxoplasma* nucleoporins. Our work increased the number of proteins that have been experimentally validated as associated with the *Toxoplasma* NPC from 8 to 23. The 6 candidates we were unable to endogenously tag include proteins with strong homology to known Nups with well-defined domains (Supplemental Figure S2-S3, Table S5), suggesting that these unvalidated proteins include additional bona fide Nups that merit future study. We have combined our predictions of domain architecture and potential homology (Supplemental S2–3, Table S5) with our phenotypic data to suggest subcomplex membership for each of our validated or well-predicted but as yet unvalidated Nups (Figure 6). As the NPC in other organisms is comprised of ~30 proteins [22,23], it is likely that our list now approaches the full complement of nucleoporins in *Toxoplasma*. Surprisingly, only 2 of the 11 validated NPC components in *Plasmodium berghei* share significant homology with any protein in *Toxoplasma.* In fact, we were unable to find potential orthologs to most *Toxoplasma* nucleoporins in *Plasmodium* (Supplemental Table S5), highlighting the level of divergence even within phylum Apicomplexa.

By examining markers for subcomplexes on opposite sides of the nuclear envelope, we identified *Toxoplasma* proteins required for the stability of specific structures within the NPC (Figures 5, 6). This analysis has allowed us to tentatively assign membership of three proteins to specific subcomplexes within the NPC. We found that degradation of TG*_203780 resulted in the specific detachment of Nup302C from the NPC, suggesting that TG*_203780 is a critical component of the Y-shaped complex, and perhaps an ortholog of ScNup1. Because ScNup1 is also part of the nuclear basket in yeast, TG*_203780 may provide a useful hook to examine what residual structure of the nuclear basket is preserved in *Toxoplasma*. We also found that two Nups, TG*_275490 and TG*_313430 are likely components of the adapter nucleoporin complex, as they are required for the stability of structures found on either side of the nuclear envelope. Intriguingly, knockdown of these putative adapter Nups does not immediately block replication; parasites are able to divide 1–2 times before dying. This is consistent with the incomplete penetrance of the NPC destabilization phenotype we observed after 8 h post-knockdown of both proteins. Taken together, these data are consistent with the idea that TG*_275490 and TG*_313430 are required for efficient assembly of the *Toxoplasma* NPC, rather than maintaining the stability of already formed complexes. Indeed, loss of function of the yeast adapter proteins ScNup157 and ScNup170 [57] results in the gradual depletion of functional NPCs with each cellular division. Thus our data suggest that the underlying paradigm of NPC assembly is conserved in *Toxoplasma* in spite of its divergent architecture. Note that ScNup157/170 are paralogs. Deletion of either gene does not affect yeast growth, though the double mutant is synthetic lethal [57]. Based on homology and secondary structure prediction (Supplemental Figure S2, Table S5), we predict that TG*_258970 is an ortholog of these proteins and found it is non-essential. It is therefore possible that a second protein in *Toxoplasma* assists TG*_258970 in its function.

We also identified the core scaffolds of both the cytoplasmic and nucleoplasmic components for mRNA export. We found clear orthologs for many components of the cytoplasmic mRNA export platform in *Toxoplasma* (Figures 4 and 6). On the other hand, the components of the nucleoplasmic TREX-2 complex have not been well conserved in *Toxoplasma*; we were able to identify and validate only the central scaffold protein Sac3 (TG*_211700) as a bona fide component of this complex. We identified potential *Toxoplasma* orthologs of two proteins that tether the TREX-2 complex the NPC in yeast, ScNup1 [39] and ScCdc31/Centrin-3 [53]. However, unlike the phenotypes in yeast, loss of the *Toxoplasma* proteins had no effect on mRNA export (Figure 4). This can be partially explained by the fact that ScNup1 serves in yeast to anchor TREX-2 to the nuclear basket, a structure that is largely missing in *Toxoplasma* [7]. These findings are in stark contrast to a recent analysis of the NPC in another divergent parasite from a different phylum, *Trypanosoma brucei*. Clear orthologs to many yeast nucleoporins have been identified in trypanosomes, including the components of the nuclear basket [15,34]. Both the cytoplasmic-facing and nuclear mRNA export machinery also appears well conserved in trypanosomes, including each of the components of the TREX-2 complex [34]. It therefore seems that any apicomplexan specialization of these complexes is unusual to the phylum.

The subcomplexes of the NPC were originally characterized biochemically – by identifying proteins that co-immunoprecipitated in different conditions. The tremendous strides in our understanding of the NPC three-dimensional structure has required an integrative approach, which has combined high-resolution structures of individual subcomplexes, crosslinking-mass spectrometry, and cryo-electron microscopy and tomography of intact NPCs [6,7,17,58–62]. All of these experiments, as with the reverse genetics that has enabled functional characterization of the NPC’s diverse functions, have required knowledge of the proteins involved. As we have now validated a large number of *Toxoplasma* Nups, we expect our data to empower future studies of the peculiarities of the structure and cellular functions of the NPC in this divergent organism.

## Materials and Methods

### PCR and Plasmids:

The primers used in this study for generation of plasmids are listed in Table S1. All PCRs were done using Phusion polymerase (NEB; New England Biolabs) and plasmids were assembled using Gibson master mix (NEB).

### Parasite Culture and transfections:

*Toxoplasma* tachyzoites were maintained on confluent HFF (Human foreskin fibroblasts) monolayers. The HFFs were cultured in DMEM (Dulbecco’s modified Eagle’s medium) supplemented with 10% FBS (fetal bovine serum) and 2 mM glutamine. The Nup302^BioID2−3xHA^, Nup134^BioID2−3xHA^ and Nup68^BioID2−3xHA^ parasites were generated by transfecting ~30 μg of linearized plasmid containing ~1500 bp of targeting sequence in frame with the BioID2 and 3xHA tags in the *RHΔku80Δhxgprt* strain [63]. Selection was performed using 25 μg/mL mycophenolic acid and 50 μg/mL xanthine (MPA/Xanthine) in the culture medium. Clonal parasite lines were obtained by infecting a 96 well plate with 1 parasite per well and screening by immunofluorescence (IFA) for HA signal using anti-HA antibody (3F10, Roche/Sigma). For generating parasites with NPC markers tagged, linearized plasmid containing homologous regions of either TgNup302, TG*_297830, or TG*_211700 in frame with 6xTy1 tag were transfected into the *RHΔku80Δhxgprt* parasites expressing OsTir1 driven by the *gra1* promoter. Selection was performed with Zeocin (Bleomycin). Briefly, parasites from a highly infected T25 were released using a syringe and a 27 gauge needle, filtered and centrifuged at 300xg to collect parasites. The parasites were resuspended in 500 μL of HBSS (Hank’s balanced salt solution) containing 25 μM Zeocin. The parasites were incubated at 37°C for 1 hour and passed onto a fresh T25 of HFFs for outgrowth before cloning by limited dilution, as above. The AID-3xHA-tagged strains for all selected candidates were generated by transfecting the *RHΔku80Δhxgprt* Ostir1/Nup302C^6xTy1^ strain with linearized plasmids containing ~700 to ~1500 bp of targeting sequence in frame with the 3xHA and AID tags. These strains were used for initial verification of candidates and determining essentiality of the candidate proteins. Essential Nups were also tagged in the background of OsTIR1/TG*_297830^6xTy1^. The Ostir1/TG*_211700^6xTy1^ strain was transfected with linearized plasmid to tag TG*_297830 with 3xHA-AID tag.

### Proximity Biotinylation and mass spectrometry:

Nup302-BioID2–3xHA, Nup134-BioID2–3xHA and Nup68-BioID2–3xHA parasites were passed into 2× 15cm dishes each and grown in presence of 150 μM biotin (Sigma-Aldrich) for 40 h. The infected HFF monolayer was washed with Phosphate Buffered Saline (PBS) 3× to remove any extracellular biotin. The parasites were released from the host cell using a syringe and a 27 gauge needle and harvested by centrifugation. The parasite pellet was resuspended and washed 3× with 50 mL PBS. The parasite pellet was then resuspended in 2.5 mL of 1x RIPA buffer and incubated at 4°C for 2 hours to ensure lysis. In order to remove remaining free biotin, each sample was buffer exchanged 2× using a pre-equilibrated PD-10 Desalting column (GE Healthcare). The biotinylated proteins were separated from the lysate using magnetic streptavidin resin (NEB). The resin was washed 2× with RIPA buffer, 1× with 20 mM Tris-HCl pH 7.5, 1 % SDS, 1× with each: 10 mM Tris-HCl pH 8.1, 250 mM LiCl, 0.5% NP40, 0.5% Sodium deoxycholate, 1 mM EDTA; 50 mM Tris-HCl pH 7.4, 50 mM NaCl, and finally 2× with PBS. Biotinylated proteins were eluted with 60 mM Tris-HCL pH 6.8, 4% SDS, 16.6% Glycerol and 10 mM β-Mercaptoethanol (2x SDS loading dye). Elutions were loaded on precast Mini-PROTEAN TGX Stain-Free Precast Gels (BioRad) and run at 200V for 6 mins. The gel was stained with gel code blue (invitrogen) and stained bands were cut and processed for mass spectrometry analysis.

For mass spectrometry analysis, individual samples were digested overnight with trypsin (Pierce) following reduction and alkylation with DTT and iodoacetamide (Sigma–Aldrich). The samples then underwent solid-phase extraction cleanup with an Oasis HLB plate (Waters) and the resulting samples were injected onto an Orbitrap Fusion Lumos mass spectrometer coupled to an Ultimate 3000 RSLC-Nano liquid chromatography system. Samples were injected onto a 75 um i.d., 50-cm long EasySpray column (Thermo) and eluted with a gradient from 0–28% buffer B over 60 min at 250 nL/min. Buffer A contained 2% (v/v) ACN and 0.1% formic acid in water, and buffer B contained 80% (v/v) ACN, 10% (v/v) trifluoroethanol, and 0.1% formic acid in water. The mass spectrometer operated in positive ion mode with a source voltage of 2.2 kV and an ion transfer tube temperature of 275 °C. MS scans were acquired at 120,000 resolution in the Orbitrap and up to 10 MS/MS spectra were obtained in the ion trap for each full spectrum acquired using higher-energy collisional dissociation (HCD) for ions with charges 2–7. Dynamic exclusion was set for 25 s after an ion was selected for fragmentation.

Raw MS data files were analyzed using Proteome Discoverer v2.2 (ThermoFisher Scientific), with peptide identification performed using Sequest HT searching against the *Toxoplasma* GT1 and ME49 proteomes from ToxoDBv68 [64]. Fragment and precursor tolerances of 10 ppm and 0.6 Da were specified, and three missed cleavages were allowed. Carbamidomethylation of Cys was set as a fixed modification, with oxidation of Met, methylation of Lys and Arg, demethylation of Lys and Arg, trimethylation of Lys, acetylation of Lys, and phosphorylation of Ser, Thr, and Tyr set as a variable modification. The false-discovery rate (FDR) cutoff was 1% for all peptides.

### Immunofluorescence and Microscopy:

HFFs were plated on 24 well plates containing 1.2 mm (diameter) glass coverslips and grown to confluency. The confluent coverslips were infected with parasites and incubated overnight or to a fixed time-point. The infected coverslips were washed with PBS and fixed with 4% paraformaldehyde + 4% sucrose in PBS at room temperature for 15 min. The coverslips were washed 3× with PBS before adding 0.1% Triton X-100 in PBS for 20 min to permeabilize the cells. The coverslips were washed 3× with PBS and blocked for 45 min with 3% Bovine Serum Albumin (BSA) in PBS in a humidity chamber. The coverslips were then incubated with primary antibody overnight at 4°C and washed 3× with 0.1% Triton X-100 in PBS with 5 min incubation between each wash. The coverslips were then treated with secondary antibody conjugated with Alexa-flour (Molecular probes) for 40 min and washed 3×, with 5 min incubation between each wash. The coverslips were then treated with Hoechst for 15 min where needed, washed with PBS and then mounted on a glass slide using mounting medium (Vector Laboratories). The cells were imaged on either a Nikon A1 laser scanning confocal microscope with a 60× oil immersion 1.42 NA objective or with a Nikon Ti2E wide-field microscope with a 100× oil immersion 1.45 NA objective using Nikon Elements. Primary antibodies used in this study are rat anti-HA (Sigma-Aldrich; 1:1,000 dilution), rabbit anti-Tg-β-tubulin (1:10,000 dilution), mouse anti-Ty1 (Sigma-Aldrich; 1:10,000). The secondary antibodies used in the study are Goat anti-Rabbit IgG, Alexa Fluor^™^ 647 (Thermo; # A-21245), Goat anti-Mouse IgG, Alexa Fluor^™^ 555 (Thermo, # A21424) and Goat anti-Rat IgG, Alexa Fluor^™^ 488 (Thermo, #A-21208).

### Plaque Assays:

To measure the survivability of parasite upon knockdown of candidate Nups, 200 or 100 parasites from syringe released T25 of a respective clonal population were added to a monolayer of HFFs in a 6-well plate in IAA or vehicle (500 μg/mL; dissolved in 100% ethanol). After 8 or 10 days, the HFF monolayer was fixed with methanol and stained with crystal violet. All plaque assays were performed in n=3 biological replicates with n=3 technical replicate per biological replicate. For the candidate Nups that showed plaques upon knockdown, the plaque areas were measured using ImageJ [65]. Significance was determined by unpaired two-tailed Student’s t-test.

### Parasite Replication and parasitophorous vacuole (PV) size measurement:

Parasite replication was quantified by counting the number of parasites per vacuole. Parasites with AID-tagged Nups were passed on coverslips and allowed to infect for 2 hours. The media was changed to ±IAA after 2 hours of infection and the coverslips were fixed 16 hours after media change. IFA was performed as above and the parasites were visualized with anti-HA, anti-Tub, anti-Ty1 antibodies, and Hoechst stain. The resulting images were analyzed using Fiji/ImageJ [65]. For each replicate, 100 vacuoles per replicate were analyzed to count the number of vacuoles containing 1, 2, 4, or 8+ parasites in ±IAA conditions for a total of n=3 biological replicates. Significance between the ±IAA conditions was determined by Kolmogorov–Smirnov test.

The number of inviable parasites was measured based on the tubulin staining for TG*_313430^AID^, TG*_203780^AID^, TG*_275490^AID^, and TG*_214600^AID^ at 24 h. The vacuole size was measured for TG*_214600^AID^ at 24h and 36h. For both measurements, the coverslips with confluent HFF monolayers were infected with clonal parasite line with a MOI of 0.1 and allowed to infect for 2 hours before changing the media to ±IAA. The coverslips were fixed at the timepoints mentioned for the respective experiments. The parasites were stained with anti-Gra1, anti-tubulin, and Hoechst stain. Maximum intensity projections of widefield images were used for measuring the vacuole size and for accessing the inviable parasites. The tubulin signal was manually assessed as normal or abnormal for n=30 parasites per biological replicate. Data from three biological replicate were plotted as normalized stacked bar graph and significance between the ±IAA conditions was calculated by Kolmogorov–Smirnov test. For measuring the vacuole size, the largest dimension of GRA1 signal was measured in ImageJ [65] for each time point. Data from three biological replicates, each an average of n>20 vacuoles, were plotted on a graph against their respective time points. Significance was calculated using unpaired two-tailed Student’s t-test at each time point.

### mRNA export assays:

We adapted a published fluorescence *in-situ* hybridization protocol [66]. To measure the defect in mRNA transport, parasites from a clonal population of AID tagged candidate parasites were allowed to infect a coverslip with a confluent HFF monolayer. The parasites were allowed to grow for 16 hours before changing the media to ±IAA for 2 hours. The HFF monolayer was fixed and permeabilized as mentioned in the Methods section (Immunofluorescence and Microscopy). Following permeabilization, the cells were then incubated in a 1:1 mixture of PBSTx (1xPBS + 0.3% Triton-X) and Pre-Hybe buffer (50% De-ionized Formamide [Roche], 5x SSC [saline-sodium citrate], 1mg/ml yeast RNA [Sigma], 1% Tween-20 [Sigma, from 10% stock]) for 10mins at RT. After transferring the coverslips to a humidity chamber, the cells were incubated in Pre-Hybe buffer for 30 min at 37°C. Post incubation, 200 ng of fluorescein tagged oligo(dT) (60-mer) mixed in Hybe buffer (50% De-ionized Formamide [Roche], 10% Dextran Sulfate [Sigma], 5x SSC, 1mg/ml yeast RNA [Sigma], 1% Tween-20 [Sigma, from 10% stock]) was added to each coverslip and further incubated overnight at 37°C. Cells were then washed with pre-warmed Wash Hybe buffer (25% Standard Formamide [Roche], 3.5x SSC, 0.5% Tween-20 [Sigma], 0.05% Triton X100 [Sigma]) 2× for 15 min each at 37°C. The cells were further washed ad additional 10× with pre-warmed 1xPBS for 10 min each at 37°C. The cells were further stained with Hoechst for 15 min at RT before mounting and imaging. The cells were imaged on a Nikon A1 laser scanning confocal microscope with a 60× oil immersion 1.42 NA objective by using Nikon Elements software. A single slice of a confocal image from the center (in z) of the nucleus was used to quantify the poly(dT) signal. Hoechst signal was used to identify nuclei. Nuclear mean fluorescein intensity was quantified from background subtracted images. N=6 biological replicates were quantified, each with a minimum n=20 technical replicates. Significance was calculated by unpaired two-tailed Student’s t-test.

### NPC marker mislocalization assay:

We checked for the NPC marker mislocalization for all the essential Nups with both TgNup302C^6xTy1^ and TG*_297830^6xTy1^ NPC markers. Clonal parasites were passed on confluent HFF monolayer growing on coverslips and the media was changed to ±IAA after 2 hours. Coverslips were fixed after 8h of ± IAA treatment. The parasites were visualized with anti-HA, anti-Tub, anti-Ty1 antibodies, and Hoechst stain. For making the scoring rubric of phenotypes and assessing the mislocalization of both NPC markers, we used maximum intensity projections of widefield images having the Ty1 and tubulin signal. 3 images per essential Nup per NPC marker per condition were used for making the rubric. The remaining images were blinded and 7 images per strain per NPC marker per condition were assessed for NPC marker localization defects for an n=5 biological replicates. For testing if there was NPC marker mislocalization (TgNup302C^6xTy1^, TG*_297830^6xTy1^, and TG*_211700^6xTy1^) in the candidates that showed mRNA accumulation (TG*_211700 and TG*_297830) upon their knockdown, we incubated the parasites in ±IAA media for 2 hours post 2 hour infection in normal media. The same strategy was used to access the NPC marker mislocalization at 2 hours for an n=3 biological replicates. The significance was calculated by Welch’s t-test and the p-values were adjusted for multiple tests using Benjamini–Hochberg false discovery rate correction.

### Bioinformatic and structural analysis:

*Toxoplasma gondii* (GT1) and *Plasmodium berghei* (ANKA) Nup sequences were obtained from VEuPathDB [64] and the yeast sequences were obtained from Uniprot [67]. BLAST [35] and PANTHER [36] searches were performed on the NCBI webserver with an e-value cutoff of 10. Reciprocal BLAST searches were performed for all hits of *Toxoplasma* and *Plasmodium* searches below a cutoff of 1e-2. For the Foldseek [37] searches, Alphafold models of *Toxoplasma*, *Plasmodium* and yeast were downloaded from the Alphafold database [68]. If the models were not available in the database, Nup sequences were used to construct models using AlphaFold3 webserver [68]. These models were used to search for orthologs on the Foldseek webserver. The secondary structure prediction was performed using s4pred [70]. The coiled-coil prediction was performed using Deepcoil2 [71]. Transmembrane domain prediction was performed using DeepTMHMM [72].

We used ChimeraX [73] to visualize, analyze and generate images showing the cryo-ET maps from *Toxoplasma* (EMD-44381), Yeast (EMD-44377) and the integrative models (PDB: 9A8M, 9A8N, 9A0F) [6,7]. We generated the TREX-2 complex model using AlphaFold 3 webserver [69]. The stoichiometries for the TREX-2 complex components were based on PDB: 4MBE (yeast Sac3:Sus1:Cdc31:Nup1 complex) [49], 3FWC (yeast Sac3:Sus1:Cdc31 complex) [74] and 8U8D (yeast Sac3:Thp1:Sem1 complex) [50]. ChimeraX was used to visualize the model and generate image for the figure.

To predict the subcomplex membership in Figure 6, we combined the above bioinformatic data with the following rubric: (i) membrane Nups are defined by one or more transmembrane domains. (ii) Central channel Nups have an FG repeat region and a helical coiled-coil region. (iii) The adapter Nups and the Y-shaped complex include proteins with an α-solenoid fold. In addition, the adapter Nups, the Y-shaped complex, and the cytoplasmic mRNA export platform include Nups containing a β-propeller followed by a helical solenoid (in the adapter and Y-shaped complex) or coiled-coil domains (mRNA export platform). (iv) Linker Nups are predicted to be intrinsically disordered and usually contain FG repeats. (v) Most components of the nuclear basket are intrinsically disordered, other than Mlp1/2 which are coiled-coil proteins.

### Figure generation:

All graphs were plotted using the matplotlib and seaborn modules in Python. Statistics were calculated using scipy.stats. All microscopy images were generated using Fiji/ImageJ [65]. All images were annotated and organized using Inkscape v1.4 to generate final figures.

## Supplementary Material

Supplement 1

## Figures and Tables

**Table 1:** Overview of predicted *Toxoplasma* NPC components.

Accession	Nup	Localization	Predicted	Validated	Predicted subcomplex
TGGT1_201700	Sec13	Unvalidated	[20,24]		Y-shaped
TGGT1_203780	Nup145	NPC	[18]	†	Nuclear basket
TGGT1_211700	SAC3	NPC	†	†	TREX-2
TGGT1_214600	Nup433	NPC	†	†	Adapter or Y-shaped
TGGT1_219120	Nup115	NPC	[20]	[20]	Unknown
TGGT1_223760	Nup169	NPC	†	†	Unknown
TGGT1_227060	Nup139a	NPC	†	†	Unknown
TGGT1_228100	-	Unvalidated	[18,20]		Unknown
TGGT1_240510	Nup134	NPC	[20]	[20]	Adapter
TGGT1_250700	Nup161	Unvalidated	†		Unknown
TGGT1_258970	Nup327	NPC	[21], †	[21], †	Adapter
TGGT1_259640	Nup302	NPC	[20]	[20]	Linker/Y-shaped
TGGT1_261430	Nup117	Unvalidated	†		
TGGT1_265840	Nup148	NPC	†	†	Nuclear basket
TGGT1_267650	Nup324	NPC	†	†	Adapter or Y-shaped
TGGT1_269290	Nup503	NPC	[20]	[20]	Adapter or Y-shaped
TGGT1_272350	RAE1/Gle2	Unvalidated	[20]		mRNA export
TGGT1_273850	Nup68	NPC	[20,24]	†	Central channel/mRNA export
TGGT1_275490	Nup216	NPC	[20]	†	Adapter
TGGT1_276890	Nup129	NPC	[20]	[20]	???
TGGT1_281640	-	Unvalidated	†		Unknown
TGGT1_286230	Nup279	NPC	†	†	Adapter or Y-shaped
TGGT1_297745	Nup413	NPC	†	†	Membrane
TGGT1_297830	Nup139b	NPC	†	†	mRNA export
TGGT1_305790	Nup206	Unvalidated	[18]		Unknown
TGGT1_306560	Nup67	NPC	[20]	[20]	Central channel
TGGT1_309250	Nup407	NPC	[20]	[20]	Unknown
TGGT1_310610	Nup43	NPC	[18]	†	
TGGT1_313430	Nup593	NPC	[18,20]	†	Adapter
TGGT1_248500	(Nup37)	Nucleus/Cytoplasm	[20,24]	†	(No NPC localization)
TGGT1_311625	-	Nucleus	[20]	†	(No NPC localization)

† Nup names are based on orthology (when non-Nup designated) or molecular weight (per the standard Nup nomenclature). Experimentally validated localizations are indicated as well as the study that demonstrated the localization (, present study). Our final prediction of subcomplex membership based on homology and functional characterization.

## Data Availability

Mass spectrometry data are available from the MassIVE database with accession MSV000098791.

## References

[1] Jamali T, Jamali Y, Mehrbod M, Mofrad MRK. Nuclear pore complex: biochemistry and biophysics of nucleocytoplasmic transport in health and disease. Int Rev Cell Mol Biol. 2011;287: 233–286. doi:10.1016/B978-0-12-386043-9.00006-2 21414590

[2] Beck M, Hurt E. The nuclear pore complex: understanding its function through structural insight. Nat Rev Mol Cell Biol. 2017;18: 73–89. doi:10.1038/nrm.2016.147 27999437

[3] Makarov AA, Padilla-Mejia NE, Field MC. Evolution and diversification of the nuclear pore complex. Biochem Soc Trans. 2021;49: 1601–1619. doi:10.1042/BST20200570 34282823 PMC8421043

[4] Lin DH, Hoelz A. The Structure of the Nuclear Pore Complex (An Update). Annu Rev Biochem. 2019;88: 725–783. doi:10.1146/annurev-biochem-062917-011901 30883195 PMC6588426

[5] Mosalaganti S, Kosinski J, Albert S, Schaffer M, Strenkert D, Salomé PA, In situ architecture of the algal nuclear pore complex. Nat Commun. 2018;9: 2361. doi:10.1038/s41467-018-04739-y 29915221 PMC6006428

[6] Allegretti M, Zimmerli CE, Rantos V, Wilfling F, Ronchi P, Fung HKH, In-cell architecture of the nuclear pore and snapshots of its turnover. Nature. 2020;586: 796–800. doi:10.1038/s41586-020-2670-5 32879490

[7] Singh D, Soni N, Hutchings J, Echeverria I, Shaikh F, Duquette M, The molecular architecture of the nuclear basket. Cell. 2024;187: 5267–5281.e13. doi:10.1016/j.cell.2024.07.020 39127037 PMC11416316

[8] Bui KH, von Appen A, DiGuilio AL, Ori A, Sparks L, Mackmull M-T, Integrated structural analysis of the human nuclear pore complex scaffold. Cell. 2013;155: 1233–1243. doi:10.1016/j.cell.2013.10.055 24315095

[9] Kosinski J, Mosalaganti S, von Appen A, Teimer R, DiGuilio AL, Wan W, Molecular architecture of the inner ring scaffold of the human nuclear pore complex. Science. 2016;352: 363–365. doi:10.1126/science.aaf0643 27081072 PMC8926079

[10] Lin DH, Stuwe T, Schilbach S, Rundlet EJ, Perriches T, Mobbs G, Architecture of the symmetric core of the nuclear pore. Science. 2016;352: aaf1015. doi:10.1126/science.aaf1015 27081075 PMC5207208

[11] Kosova B, Panté N, Rollenhagen C, Hurt E. Nup192p is a conserved nucleoporin with a preferential location at the inner site of the nuclear membrane. J Biol Chem. 1999;274: 22646–22651. doi:10.1074/jbc.274.32.22646 10428845

[12] Grandi P, Doye V, Hurt EC. Purification of NSP1 reveals complex formation with “GLFG” nucleoporins and a novel nuclear pore protein NIC96. EMBO J. 1993;12: 3061–3071. doi:10.1002/j.1460-2075.1993.tb05975.x 7688296 PMC413571

[13] Guan T, Müller S, Klier G, Panté N, Blevitt JM, Haner M, Structural analysis of the p62 complex, an assembly of O-linked glycoproteins that localizes near the central gated channel of the nuclear pore complex. Mol Biol Cell. 1995;6: 1591–1603. doi:10.1091/mbc.6.11.1591 8589458 PMC301313

[14] Fontoura BM, Blobel G, Matunis MJ. A conserved biogenesis pathway for nucleoporins: proteolytic processing of a 186-kilodalton precursor generates Nup98 and the novel nucleoporin, Nup96. J Cell Biol. 1999;144: 1097–1112. doi:10.1083/jcb.144.6.1097 10087256 PMC2150585

[15] DeGrasse JA, DuBois KN, Devos D, Siegel TN, Sali A, Field MC, Evidence for a shared nuclear pore complex architecture that is conserved from the last common eukaryotic ancestor. Mol Cell Proteomics MCP. 2009;8: 2119–2130. doi:10.1074/mcp.M900038-MCP200 19525551 PMC2742445

[16] Fernandez-Martinez J, Rout MP. One Ring to Rule them All? Structural and Functional Diversity in the Nuclear Pore Complex. Trends Biochem Sci. 2021;46: 595–607. doi:10.1016/j.tibs.2021.01.003 33563541 PMC8195821

[17] Kim SJ, Fernandez-Martinez J, Nudelman I, Shi Y, Zhang W, Raveh B, Integrative structure and functional anatomy of a nuclear pore complex. Nature. 2018;555: 475–482. doi:10.1038/nature26003 29539637 PMC6022767

[18] Kehrer J, Kuss C, Andres-Pons A, Reustle A, Dahan N, Devos D, Nuclear Pore Complex Components in the Malaria Parasite Plasmodium berghei. Sci Rep. 2018;8: 11249. doi:10.1038/s41598-018-29590-5 30050042 PMC6062611

[19] Ambekar SV, Beck JR, Mair GR. TurboID Identification of Evolutionarily Divergent Components of the Nuclear Pore Complex in the Malaria Model Plasmodium berghei. mBio. 2022; e0181522. doi:10.1128/mbio.01815-22 36040030 PMC9601220

[20] Courjol F, Mouveaux T, Lesage K, Saliou J-M, Werkmeister E, Bonabaud M, Characterization of a nuclear pore protein sheds light on the roles and composition of the Toxoplasma gondii nuclear pore complex. Cell Mol Life Sci. 2017;74: 2107–2125. doi:10.1007/s00018-017-2459-3 28138739 PMC11107709

[21] Ovciarikova J, Shikha S, Lacombe A, Courjol F, McCrone R, Hussain W, Two ancient membrane pores mediate mitochondrial-nucleus membrane contact sites. J Cell Biol. 2024;223: e202304075. doi:10.1083/jcb.202304075 38456969 PMC10923651

[22] Rout MP, Aitchison JD, Suprapto A, Hjertaas K, Zhao Y, Chait BT. The yeast nuclear pore complex: composition, architecture, and transport mechanism. J Cell Biol. 2000;148: 635–651. doi:10.1083/jcb.148.4.635 10684247 PMC2169373

[23] Cronshaw JM, Krutchinsky AN, Zhang W, Chait BT, Matunis MJ. Proteomic analysis of the mammalian nuclear pore complex. J Cell Biol. 2002;158: 915–927. doi:10.1083/jcb.200206106 12196509 PMC2173148

[24] Neumann N, Lundin D, Poole AM. Comparative genomic evidence for a complete nuclear pore complex in the last eukaryotic common ancestor. PloS One. 2010;5: e13241. doi:10.1371/journal.pone.0013241 20949036 PMC2951903

[25] Roux KJ, Kim DI, Raida M, Burke B. A promiscuous biotin ligase fusion protein identifies proximal and interacting proteins in mammalian cells. J Cell Biol. 2012;196: 801–10. doi:10.1083/jcb.201112098 22412018 PMC3308701

[26] Kim DI, Birendra KC, Zhu W, Motamedchaboki K, Doye V, Roux KJ. Probing nuclear pore complex architecture with proximity-dependent biotinylation. Proc Natl Acad Sci U S A. 2014;111: E2453–2461. doi:10.1073/pnas.1406459111 24927568 PMC4066523

[27] Branon TC, Bosch JA, Sanchez AD, Udeshi ND, Svinkina T, Carr SA, Efficient proximity labeling in living cells and organisms with TurboID. Nat Biotechnol. 2018;36: 880–887. doi:10.1038/nbt.4201 30125270 PMC6126969

[28] Kim DI, Jensen SC, Noble KA, Kc B, Roux KH, Motamedchaboki K, An improved smaller biotin ligase for BioID proximity labeling. Mol Biol Cell. 2016;27: 1188–1196. doi:10.1091/mbc.E15-12-0844 26912792 PMC4831873

[29] Otto TA, Bergsma T, Dekker M, Mouton SN, Gallardo P, Wolters JC, Nucleoporin Nsp1 surveils the phase state of FG-Nups. Cell Rep. 2024;43: 114793. doi:10.1016/j.celrep.2024.114793 39356635

[30] Almagro Armenteros JJ, Salvatore M, Emanuelsson O, Winther O, von Heijne G, Elofsson A, Detecting sequence signals in targeting peptides using deep learning. Life Sci Alliance. 2019;2: e201900429. doi:10.26508/lsa.201900429 31570514 PMC6769257

[31] Barylyuk K, Koreny L, Ke H, Butterworth S, Crook OM, Lassadi I, A Comprehensive Subcellular Atlas of the Toxoplasma Proteome via hyperLOPIT Provides Spatial Context for Protein Functions. Cell Host Microbe. 2020;28: 752–766.e9. doi:10.1016/j.chom.2020.09.011 33053376 PMC7670262

[32] Nishimura K, Fukagawa T, Takisawa H, Kakimoto T, Kanemaki M. An auxin-based degron system for the rapid depletion of proteins in nonplant cells. Nat Methods. 2009;6: 917–22. doi:10.1038/nmeth.1401 19915560

[33] Obado SO, Field MC, Rout MP. Comparative interactomics provides evidence for functional specialization of the nuclear pore complex. Nucl Austin Tex. 2017;8: 340–352. doi:10.1080/19491034.2017.1313936 PMC5597298 28463551

[34] Gabiatti BP, Krenzer J, Braune S, Krüger T, Zoltner M, Kramer S. Detailed characterisation of the trypanosome nuclear pore architecture reveals conserved asymmetrical functional hubs that drive mRNA export. PLoS Biol. 2025;23: e3003024. doi:10.1371/journal.pbio.3003024 39899609 PMC11825100

[35] Camacho C, Coulouris G, Avagyan V, Ma N, Papadopoulos J, Bealer K, BLAST+: architecture and applications. BMC Bioinformatics. 2009;10: 421. doi:10.1186/1471-2105-10-421 20003500 PMC2803857

[36] Thomas PD, Ebert D, Muruganujan A, Mushayahama T, Albou L-P, Mi H. PANTHER: Making genome-scale phylogenetics accessible to all. Protein Sci Publ Protein Soc. 2022;31: 8–22. doi:10.1002/pro.4218 PMC8740835 34717010

[37] van Kempen M, Kim SS, Tumescheit C, Mirdita M, Lee J, Gilchrist CLM, Fast and accurate protein structure search with Foldseek. Nat Biotechnol. 2024;42: 243–246. doi:10.1038/s41587-023-01773-0 37156916 PMC10869269

[38] Ellisdon AM, Dimitrova L, Hurt E, Stewart M. Structural basis for the assembly and nucleic acid binding of the TREX-2 transcription-export complex. Nat Struct Mol Biol. 2012;19: 328–336. doi:10.1038/nsmb.2235 22343721 PMC3303126

[39] Fischer T, Strässer K, Rácz A, Rodriguez-Navarro S, Oppizzi M, Ihrig P, The mRNA export machinery requires the novel Sac3p-Thp1p complex to dock at the nucleoplasmic entrance of the nuclear pores. EMBO J. 2002;21: 5843–5852. doi:10.1093/emboj/cdf590 12411502 PMC131087

[40] Wente SR, Rout MP, Blobel G. A new family of yeast nuclear pore complex proteins. J Cell Biol. 1992;119: 705–723. doi:10.1083/jcb.119.4.705 1385442 PMC2289698

[41] Wimmer C, Doye V, Grandi P, Nehrbass U, Hurt EC. A new subclass of nucleoporins that functionally interact with nuclear pore protein NSP1. EMBO J. 1992;11: 5051–5061. doi:10.1002/j.1460-2075.1992.tb05612.x 1464327 PMC556983

[42] Engel SR, Aleksander S, Nash RS, Wong ED, Weng S, Miyasato SR, Saccharomyces Genome Database: Advances in Genome Annotation, Expanded Biochemical Pathways, and Other Key Enhancements. Genetics. 2024; iyae185. doi:10.1093/genetics/iyae185 PMC11912841 39530598

[43] Hubert T, Vandekerckhove J, Gettemans J. Exo70-mediated recruitment of nucleoporin Nup62 at the leading edge of migrating cells is required for cell migration. Traffic Cph Den. 2009;10: 1257–1271. doi:10.1111/j.1600-0854.2009.00940.x 19552648

[44] Hashizume C, Moyori A, Kobayashi A, Yamakoshi N, Endo A, Wong RW. Nucleoporin Nup62 maintains centrosome homeostasis. Cell Cycle Georget Tex. 2013;12: 3804–3816. doi:10.4161/cc.26671 PMC3905072 24107630

[45] Blasius TL, Takao D, Verhey KJ. NPHP proteins are binding partners of nucleoporins at the base of the primary cilium. PloS One. 2019;14: e0222924. doi:10.1371/journal.pone.0222924 31553752 PMC6760808

[46] Endicott SJ, Brueckner M. NUP98 Sets the Size-Exclusion Diffusion Limit through the Ciliary Base. Curr Biol CB. 2018;28: 1643–1650.e3. doi:10.1016/j.cub.2018.04.014 29731308 PMC7106777

[47] Marquez J, Bhattacharya D, Lusk CP, Khokha MK. Nucleoporin NUP205 plays a critical role in cilia and congenital disease. Dev Biol. 2021;469: 46–53. doi:10.1016/j.ydbio.2020.10.001 33065118 PMC7722132

[48] de Leon JC, Scheumann N, Beatty W, Beck JR, Tran JQ, Yau C, A SAS-6-like protein suggests that the Toxoplasma conoid complex evolved from flagellar components. Eukaryot Cell. 2013;12: 1009–1019. doi:10.1128/EC.00096-13 23687115 PMC3697468

[49] Jani D, Valkov E, Stewart M. Structural basis for binding the TREX2 complex to nuclear pores, GAL1 localisation and mRNA export. Nucleic Acids Res. 2014;42: 6686–6697. doi:10.1093/nar/gku252 24705649 PMC4041426

[50] Xie Y, Clarke BP, Xie D, Mei M, Bhat P, Hill PS, Structures and mRNP remodeling mechanism of the TREX-2 complex. Struct Lond Engl 1993. 2025;33: 566–582.e6. doi:10.1016/j.str.2024.12.019 PMC11890942 39862860

[51] Hurwitz ME, Blobel G. NUP82 is an essential yeast nucleoporin required for poly(A)+ RNA export. J Cell Biol. 1995;130: 1275–1281. doi:10.1083/jcb.130.6.1275 7559751 PMC2120582

[52] Lu Q, Tang X, Tian G, Wang F, Liu K, Nguyen V, Arabidopsis homolog of the yeast TREX-2 mRNA export complex: components and anchoring nucleoporin. Plant J Cell Mol Biol. 2010;61: 259–270. doi:10.1111/j.1365-313X.2009.04048.x 19843313

[53] Fischer T, Rodríguez-Navarro S, Pereira G, Rácz A, Schiebel E, Hurt E. Yeast centrin Cdc31 is linked to the nuclear mRNA export machinery. Nat Cell Biol. 2004;6: 840–848. doi:10.1038/ncb1163 15311284

[54] Hu K, Johnson J, Florens L, Fraunholz M, Suravajjala S, DiLullo C, Cytoskeletal components of an invasion machine--the apical complex of Toxoplasma gondii. PLoS Pathog. 2006;2: e13. doi:10.1371/journal.ppat.0020013 16518471 PMC1383488

[55] Alcázar-Román AR, Bolger TA, Wente SR. Control of mRNA export and translation termination by inositol hexakisphosphate requires specific interaction with Gle1. J Biol Chem. 2010;285: 16683–16692. doi:10.1074/jbc.M109.082370 20371601 PMC2878036

[56] Murphy R, Wente SR. An RNA-export mediator with an essential nuclear export signal. Nature. 1996;383: 357–360. doi:10.1038/383357a0 8848052

[57] Makio T, Stanton LH, Lin C-C, Goldfarb DS, Weis K, Wozniak RW. The nucleoporins Nup170p and Nup157p are essential for nuclear pore complex assembly. J Cell Biol. 2009;185: 459–473. doi:10.1083/jcb.200810029 19414608 PMC2700392

[58] Fernandez-Martinez J, Phillips J, Sekedat MD, Diaz-Avalos R, Velazquez-Muriel J, Franke JD, Structure-function mapping of a heptameric module in the nuclear pore complex. J Cell Biol. 2012;196: 419–434. doi:10.1083/jcb.201109008 22331846 PMC3283990

[59] Fernandez-Martinez J, Kim SJ, Shi Y, Upla P, Pellarin R, Gagnon M, Structure and Function of the Nuclear Pore Complex Cytoplasmic mRNA Export Platform. Cell. 2016;167: 1215–1228.e25. doi:10.1016/j.cell.2016.10.028 27839866 PMC5130164

[60] Akey CW, Singh D, Ouch C, Echeverria I, Nudelman I, Varberg JM, Comprehensive structure and functional adaptations of the yeast nuclear pore complex. Cell. 2022;185: 361–378.e25. doi:10.1016/j.cell.2021.12.015 34982960 PMC8928745

[61] Akey CW, Echeverria I, Ouch C, Nudelman I, Shi Y, Wang J, Implications of a multiscale structure of the yeast nuclear pore complex. Mol Cell. 2023;83: 3283–3302.e5. doi:10.1016/j.molcel.2023.08.025 37738963 PMC10630966

[62] Alber F, Dokudovskaya S, Veenhoff LM, Zhang W, Kipper J, Devos D, The molecular architecture of the nuclear pore complex. Nature. 2007;450: 695–701. doi:10.1038/nature06405 18046406

[63] Huynh M-H, Carruthers VB. A Toxoplasma gondii Ortholog of Plasmodium GAMA Contributes to Parasite Attachment and Cell Invasion. mSphere. 2016;1. doi:10.1128/mSphere.00012-16 PMC4863602 27303694

[64] Alvarez-Jarreta J, Amos B, Aurrecoechea C, Bah S, Barba M, Barreto A, VEuPathDB: the eukaryotic pathogen, vector and host bioinformatics resource center in 2023. Nucleic Acids Res. 2024;52: D808–D816. doi:10.1093/nar/gkad1003 37953350 PMC10767879

[65] Schindelin J, Arganda-Carreras I, Frise E, Kaynig V, Longair M, Pietzsch T, Fiji: an open-source platform for biological-image analysis. Nat Methods. 2012;9: 676–682. doi:10.1038/nmeth.2019 22743772 PMC3855844

[66] Cogswell AA, Collins JJ, Newmark PA, Williams DL. Whole mount in situ hybridization methodology for Schistosoma mansoni. Mol Biochem Parasitol. 2011;178: 46–50. doi:10.1016/j.molbiopara.2011.03.001 21397637 PMC3102561

[67] UniProt Consortium. UniProt: the Universal Protein Knowledgebase in 2025. Nucleic Acids Res. 2025;53: D609–D617. doi:10.1093/nar/gkae1010 39552041 PMC11701636

[68] Varadi M, Bertoni D, Magana P, Paramval U, Pidruchna I, Radhakrishnan M, AlphaFold Protein Structure Database in 2024: providing structure coverage for over 214 million protein sequences. Nucleic Acids Res. 2024;52: D368–D375. doi:10.1093/nar/gkad1011 37933859 PMC10767828

[69] Abramson J, Adler J, Dunger J, Evans R, Green T, Pritzel A, Accurate structure prediction of biomolecular interactions with AlphaFold 3. Nature. 2024;630: 493–500. doi:10.1038/s41586-024-07487-w 38718835 PMC11168924

[70] Moffat L, Jones DT. Increasing the accuracy of single sequence prediction methods using a deep semi-supervised learning framework. Bioinforma Oxf Engl. 2021;37: 3744–3751. doi:10.1093/bioinformatics/btab491 PMC8570780 34213528

[71] Ludwiczak J, Winski A, Szczepaniak K, Alva V, Dunin-Horkawicz S. DeepCoil-a fast and accurate prediction of coiled-coil domains in protein sequences. Bioinforma Oxf Engl. 2019;35: 2790–2795. doi:10.1093/bioinformatics/bty1062 30601942

[72] Hallgren J, Tsirigos KD, Pedersen MD, Armenteros JJA, Marcatili P, Nielsen H, DeepTMHMM predicts alpha and beta transmembrane proteins using deep neural networks. bioRxiv; 2022. p. 2022.04.08.487609. doi:10.1101/2022.04.08.487609

[73] Pettersen EF, Goddard TD, Huang CC, Meng EC, Couch GS, Croll TI, UCSF ChimeraX: Structure visualization for researchers, educators, and developers. Protein Sci Publ Protein Soc. 2021;30: 70–82. doi:10.1002/pro.3943 PMC7737788 32881101

[74] Jani D, Lutz S, Marshall NJ, Fischer T, Köhler A, Ellisdon AM, Sus1, Cdc31, and the Sac3 CID region form a conserved interaction platform that promotes nuclear pore association and mRNA export. Mol Cell. 2009;33: 727–737. doi:10.1016/j.molcel.2009.01.033 19328066 PMC2726291

