# Supplementary material for: Identification of divergent Toxoplasma Nuclear Pore Complex components highlights speciation of mRNA export machinery
Source: bioRxiv. 2025 Aug 27:2025.08.27.672535. Preprint. [Version 1] doi: 10.1101/2025.08.27.672535 (PMC12407827; doi:10.1101/2025.08.27.672535)
Supplement: Supplement 1 [file NIHPP2025.08.27.672535v1-supplement-1.pdf]

bioRxiv preprint doi: https://doi.org/10.1101/2025.08.27.672535; this version posted August 27, 2025. The copyright holder for this preprint (which was not certified by peer review) is the author/funder, who has granted bioRxiv a license to display the preprint in perpetuity. It is made available under aCC-BY 4.0 International license.

Supplemental Data S1 – Mass spectrometry data from proximity biotinylation experiments. Mass spectrometry data are available from the MassIVE database with accession MSV000098791.

Supplemental Figure S2: Domain architecture of essential and annotated Nups in *Toxoplasma.* The *Toxoplasma* Nups in red (essential Nups) or blue (non-essential) are divided into groups based on the Nup type, which are shown as vertical rectangular box on the left. Below the *Toxoplasma* Nups are the predicted yeast orthologs in black marked by the prediction type (PANTHER, BLAST, or Foldseek). The proteins are indicated by rectangular boxes where the protein length is in-scale to the residue numbers at the top. The secondary structure probabilities for helix (orange) and beta sheet (blue) are graphed inside the rectangular boxes. The boxed symbols in bottom right corner describe the predicted domains marked above the protein boxes.

Supplemental Figure S3: Domain architecture of non-essential and unverified Nups in *Toxoplasma*. The *Toxoplasma* Nups in blue (non-essential Nups) or gray (unvalidated) are divided into groups based on the Nup type, which are shown as vertical rectangular box on the left. All annotations are as in Figure S2.

bioRxiv preprint doi: https://doi.org/10.1101/2025.08.27.672535; this version posted August 27, 2025. The copyright holder for this preprint (which was not certified by peer review) is the author/funder, who has granted bioRxiv a license to display the preprint in perpetuity. It is made available under aCC-BY 4.0 International license.

Supplemental Figure S4: A subset of candidates did not localize to the NPC. (A) Intracellular parasites in which the indicated candidates (for clarity, only the numerical identifiers are indicated) had been tagged with AID-3xHA were stained with antibodies recognizing HA (green), Ty1 (red; Nup302C), and *Toxoplasma* β-tubulin (not shown; used to create outline). DNA was stained with Hoechst (blue). Images are 1 airy-unit confocal slices (~0.75 μm). All scale bars are 5 μm. Note lack of localization to NPC (red) for all candidates. While some of these non-Nup candidates were unable to form plaques when grown in +IAA (B), others showed no loss in plaque efficiency (C). (D) A subset of these non-essential proteins showed mild fitness cost upon growth in +IAA, per a reduction in plaque area. ns, not significant; *, $p<0.05$; n=3 biological replicates two-tailed unpaired Student's t-test.

Supplemental Table S5 – Spreadsheet detailing search hits and corresponding e-values from BLAST, PANTHER, and Foldseek.

Supplemental Figure S6: Block in replication at late timepoints for a subset of essential Nups. (A) The percent of vacuoles showing abnormal tubulin staining after 24 h growth in ±IAA. Note that while TG*_214600 shows no quantifiable phenotype, tubulin staining does not appear entirely normal after 24 h growth in +IAA. (B) TG*_214600 AID parasites grown in +IAA showed no significant increase in vacuole size from 24-36h, suggesting a block in growth at late timepoints. All scale bars are 10 μm.

Supplemental Figure S7: Additional characterization of proteins involved in mRNA export. (A) TgGle1$^{AID-3xHA}$ localizes to the NPC (orange arrows), while TgCen3$^{AID-3xHA}$ shows expected punctate staining consistent with centrosomal localization (white arrows). (B) Both TgGle1$^{AID-3xHA}$ and TgCen3$^{AID-3xHA}$ strains are unable to form plaques when grown in +IAA. (C) Quantification of the indicated markers (Nup302C, 297830, or 211700) in TG*_211700^{AID} and TG*_297830^{AID} strains after incubation in ±IAA for 2 h. p-values (non-significant) indicated from unpaired two-tailed Student's t-test.

bioRxiv preprint doi: https://doi.org/10.1101/2025.08.27.672535; this version posted August 27, 2025. The copyright holder for this preprint (which was not certified by peer review) is the author/funder, who has granted bioRxiv a license to display the preprint in perpetuity. It is made available under aCC-BY 4.0 International license.

Supplemental Table S8: – Primer sequences used in this study.

bioRxiv preprint doi: https://doi.org/10.1101/2025.08.27.672535; this version posted August 27, 2025. The copyright holder for this preprint (which was not certified by peer review) is the author/funder, who has granted bioRxiv a license to display the preprint in perpetuity. It is made available under aCC-BY 4.0 International license.

Supplemental Data S1 – Mass spectrometry data from proximity biotinylation experiments. Mass spectrometry data are available from the MassIVE database with accession MSV000098791.

Supplemental Table S5 – Spreadsheet detailing search hits and corresponding e-values from BLAST, PANTHER, and Foldseek.

Supplemental Table S8 – Primer sequences used in this study.

bioRxiv preprint doi: https://doi.org/10.1101/2025.08.27.672535; this version posted August 27, 2025. The copyright holder for this preprint (which was not certified by peer review) is the author/funder, who has granted bioRxiv a license to display the preprint in perpetuity. It is made available under aCC-BY 4.0 International license.

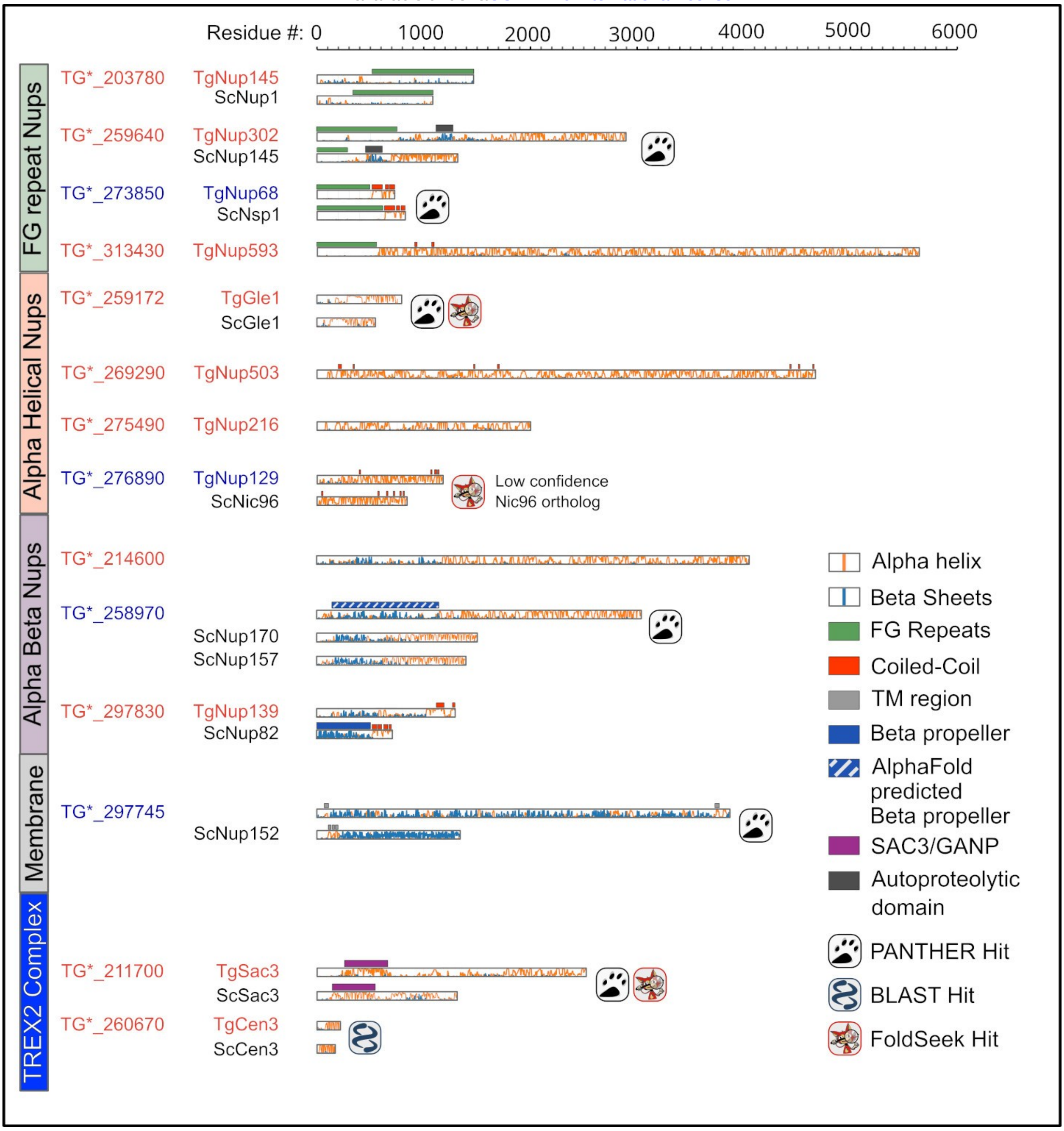

Supplemental Figure S2: Domain architecture of essential and annotated Nups in *Toxoplasma*. The *Toxoplasma* Nups in red (essential Nups) or blue (non-essential) are divided into groups based on the Nup type, which are shown as vertical rectangular box on the left. Below the *Toxoplasma* Nups are the predicted yeast orthologs in black marked by the prediction type (PANTHER, BLAST, or Foldseek). The proteins are indicated by rectangular boxes where the protein length is in-scale to the residue numbers at the top. The secondary structure probabilities for helix (orange) and beta sheet (blue) are graphed inside the rectangular boxes. The boxed symbols in bottom right corner describe the predicted domains marked above the protein boxes.

bioRxiv preprint doi: https://doi.org/10.1101/2025.08.27.672535; this version posted August 27, 2025. The copyright holder for this preprint (which was not certified by peer review) is the author/funder, who has granted bioRxiv a license to display the preprint in perpetuity. It is made available under aCC-BY 4.0 International license.

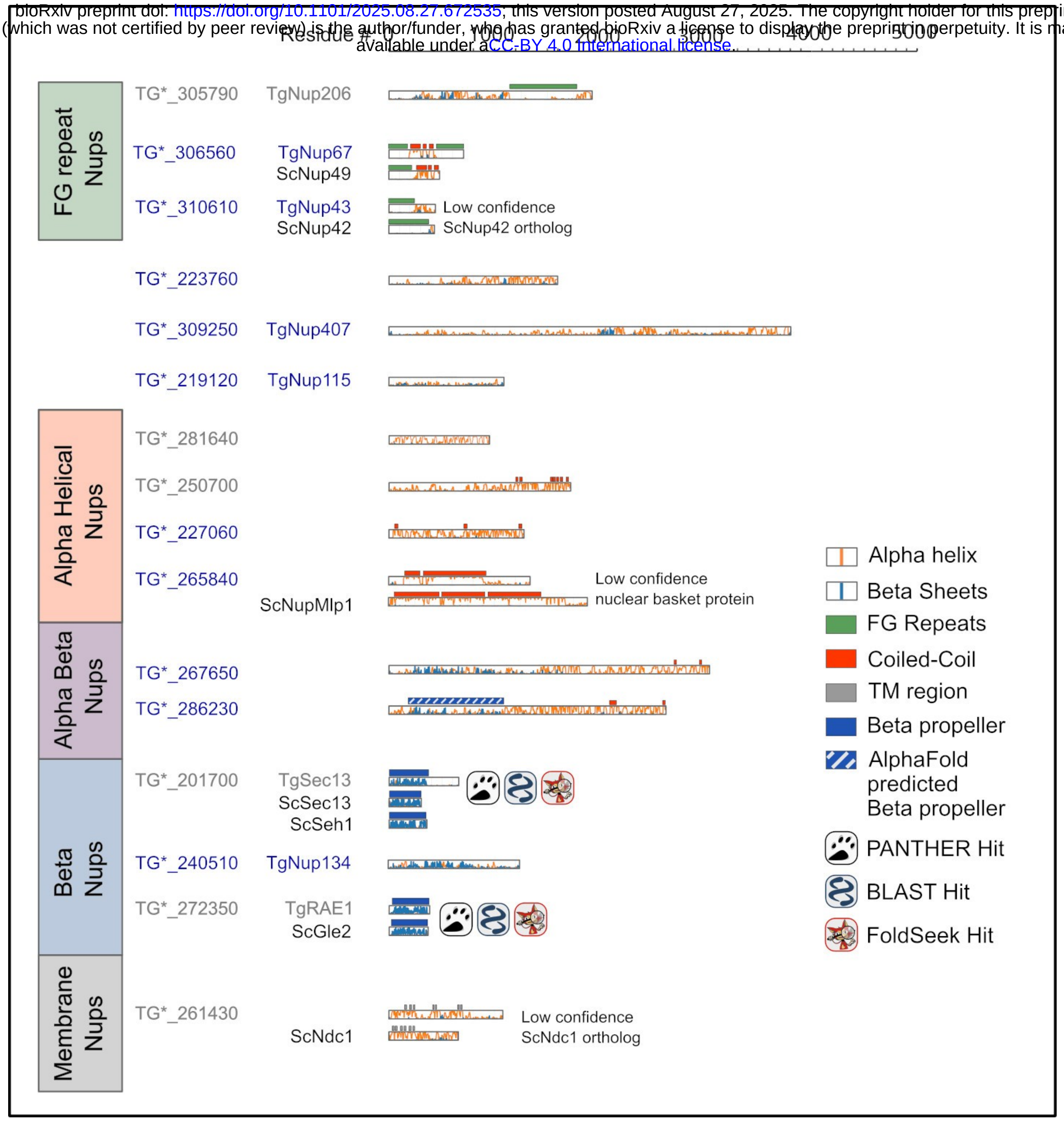

Supplemental Figure S3: Domain architecture of non-essential and unverified Nups in *Toxoplasma*. The *Toxoplasma* Nups in blue (non-essential Nups) or gray (unvalidated) are divided into groups based on the Nup type, which are shown as vertical rectangular box on the left. All annotations are as in Figure S2.

bioRxiv preprint doi: https://doi.org/10.1101/2025.08.27.672535; this version posted August 27, 2025. The copyright holder for this preprint (which was not certified by peer review) is the author/funder, who has granted bioRxiv a license to display the preprint in perpetuity. It is made available under aCC-BY 4.0 International license.

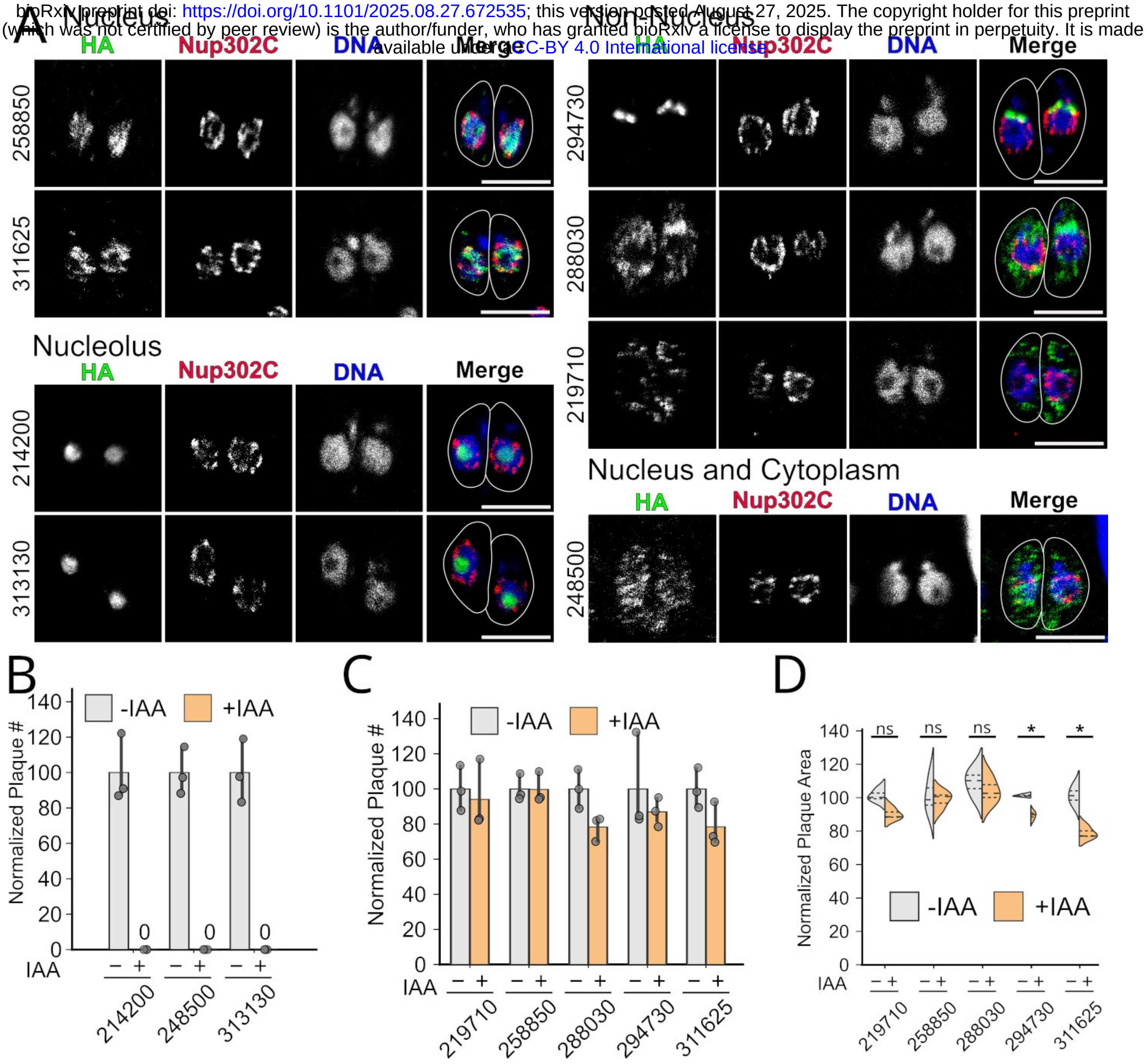

Supplemental Figure S4: A subset of candidates did not localize to the NPC. (A) Intracellular parasites in which the indicated candidates (for clarity, only the numerical identifiers are indicated, *i.e.*, TG*_######) had been tagged with AID-3xHA were stained with antibodies recognizing HA (green), Ty1 (red; Nup302C), and *Toxoplasma* β-tubulin (not shown; used to create outline). DNA was stained with Hoechst (blue). Images are 1 airy-unit confocal slices (~0.75 µm). All scale bars are 5 µm. Note lack of localization to NPC (red) for all candidates. While some of these non-Nup candidates were unable to form plaques when grown in +IAA (B), others showed no loss in plaque efficiency (C). (D) A subset of these non-essential proteins showed mild fitness cost upon growth in +IAA, per a reduction in plaque area. ns, not significant; *, $p<0.05$; n=3 biological replicates two-tailed unpaired Student's t-test.

bioRxiv preprint doi: https://doi.org/10.1101/2025.08.27.672535; this version posted August 27, 2025. The copyright holder for this preprint (which was not certified by peer review) is the author/funder, who has granted bioRxiv a license to display the preprint in perpetuity. It is made available under aCC-BY 4.0 International license.

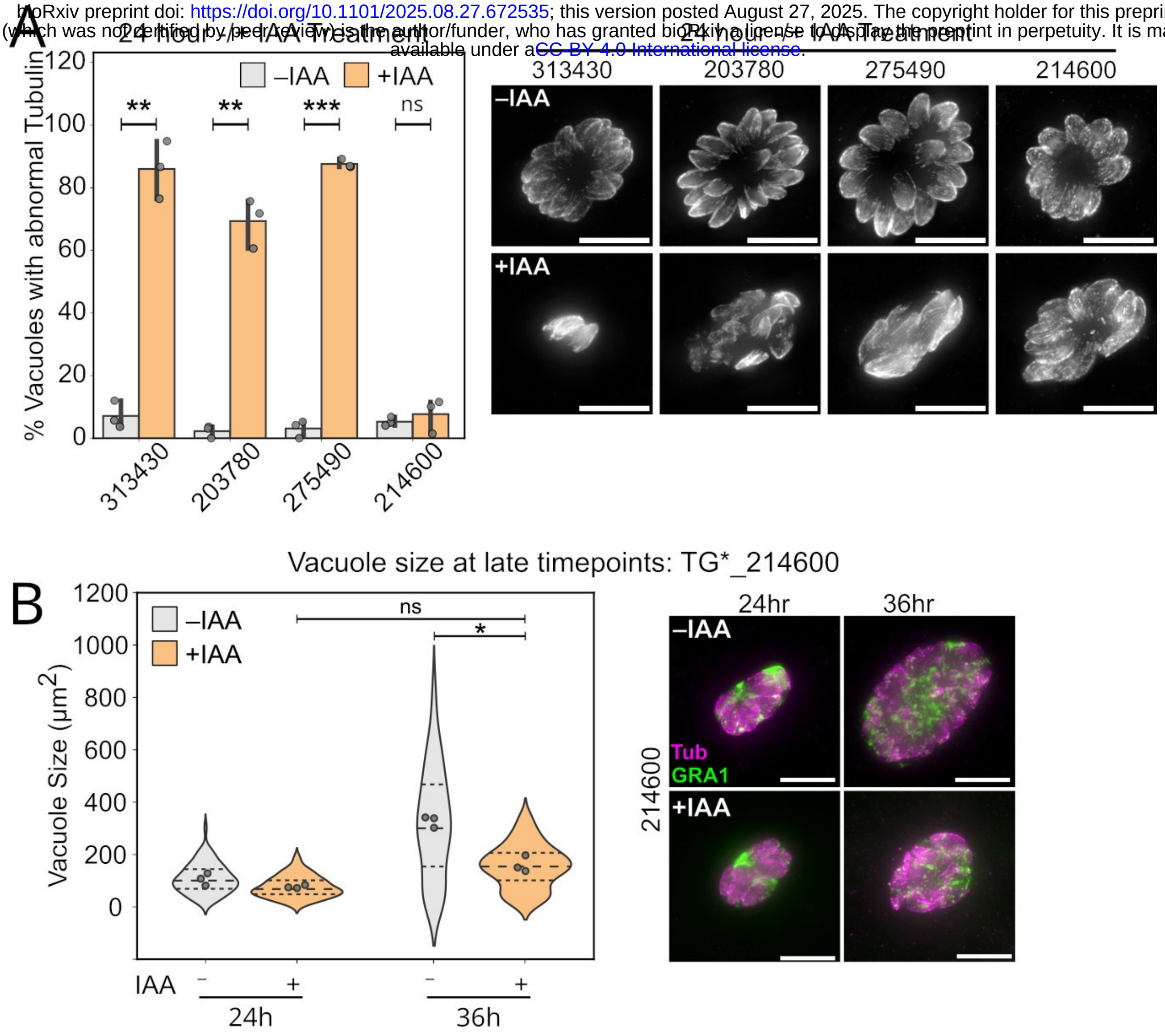

Supplemental Figure S6: Block in replication at late timepoints for a subset of essential Nups. (A) The percent of vacuoles showing abnormal tubulin staining after 24 h growth in ±IAA. Note that while TG*_214600 shows no quantifiable phenotype, tubulin staining does not appear entirely normal after 24 h growth in +IAA. (B) TG*_214600 AID parasites grown in +IAA showed no significant increase in vacuole size from 24-36h, suggesting a block in growth at late timepoints. All scale bars are 10 μm.

bioRxiv preprint doi: https://doi.org/10.1101/2025.08.27.672535; this version posted August 27, 2025. The copyright holder for this preprint (which was not certified by peer review) is the author/funder, who has granted bioRxiv a license to display the preprint in perpetuity. It is made available under a CC-BY 4.0 International license.

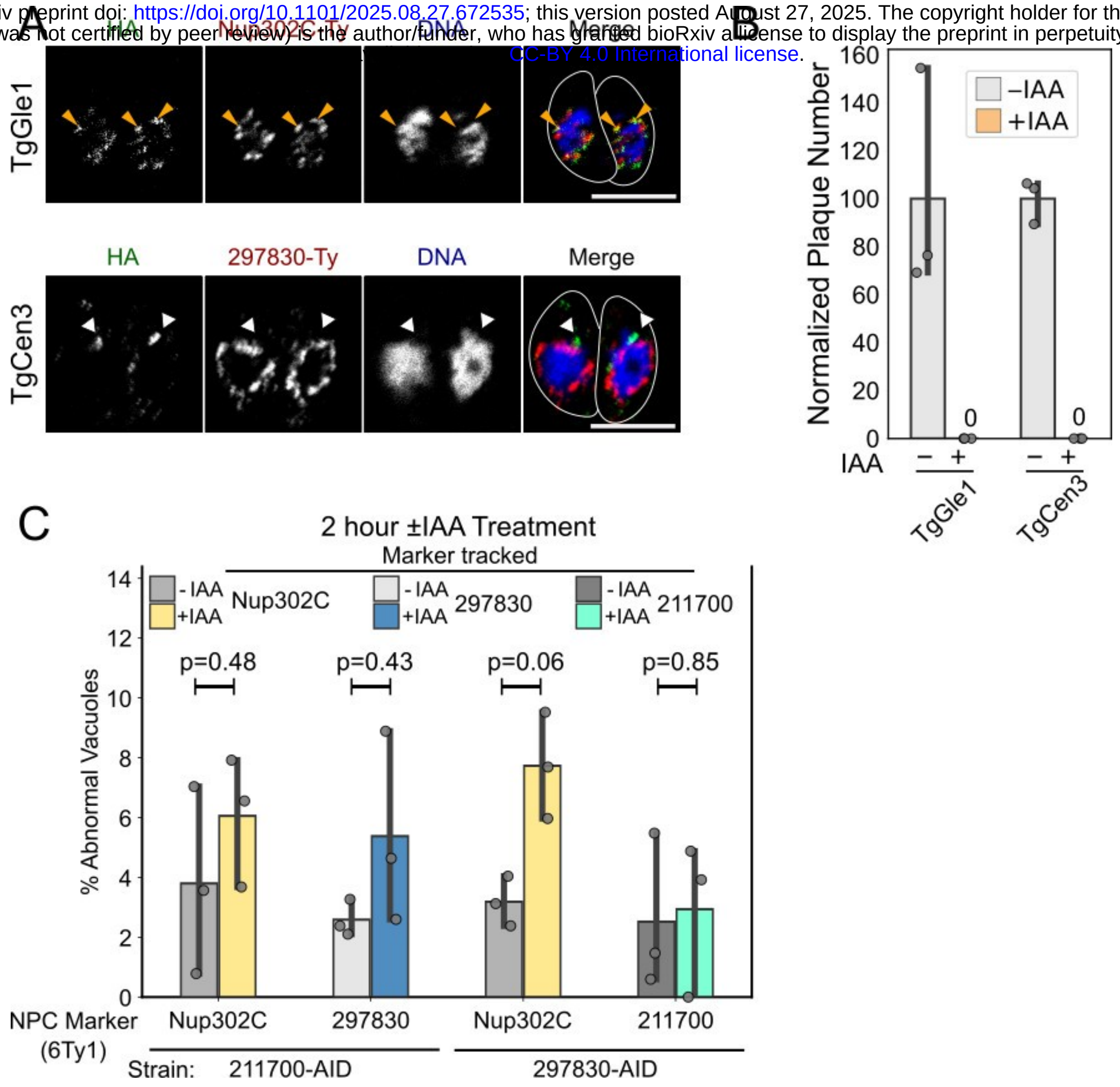

Supplemental Figure S7: Additional characterization of proteins involved in mRNA export. (A) TgGle1$^{AID\text{-}3xHA}$ localizes to the NPC (orange arrows), while TgCen3$^{AID\text{-}3xHA}$ shows expected punctate staining consistent with centrosomal localization (white arrows). (B) Both TgGle1$^{AID\text{-}3xHA}$ and TgCen3$^{AID\text{-}3xHA}$ strains are unable to form plaques when grown in +IAA. (C) Quantification of the indicated markers (Nup302C, 297830, or 211700) in TG*_211700^{AID} and TG*_297830^{AID} strains after incubation in ±IAA for 2 h. p-values (non-significant) indicated from unpaired two-tailed Student's t-test.
